# Supplementary material for: Rho-Kinase Planar Polarization at Tissue Boundaries Depends on Phospho-regulation of Membrane Residence Time
Source: Dev Cell. 2020 Feb 10;52(3):364–378.e7. doi: 10.1016/j.devcel.2019.12.003 (PMC7008249; doi:10.1016/j.devcel.2019.12.003)
Supplement: Document S1. Figures S1–S5 [file mmc1.pdf]

Developmental Cell, Volume *52*

## Supplemental Information

# Rho-Kinase Planar Polarization at Tissue Boundaries Depends on Phospho-regulation of Membrane Residence Time

**Clara Sidor, Tim J. Stevens, Li Jin, Jérôme Boulanger, and Katja Röper**

***Figure S1, related to Figure 1. Wide-spread apical planar polarisation of Crumbs and myosin II during morphogenesis.***

**A-A''** Still images of a time lapse movie of embryos expressing tagged myosin II (*Zipper-YFP*, green) and Crumbs (*Crumbs-GFP*, magenta), Fig.1A' shows t=0 min and t=99 min of this movie, intervening time points are displayed here to illustrate the morphogenetic process.

**B-E'** mNG-Rok (green in composites) and myosin regulatory light chain (labeled using *sqh-mCherry*; magenta in composites) colocalise in their accumulation across embryonic tissues: **B-C'** salivary gland placode, **D-D'** posterior spiracles, **E-E'** stage 15 epidermis.

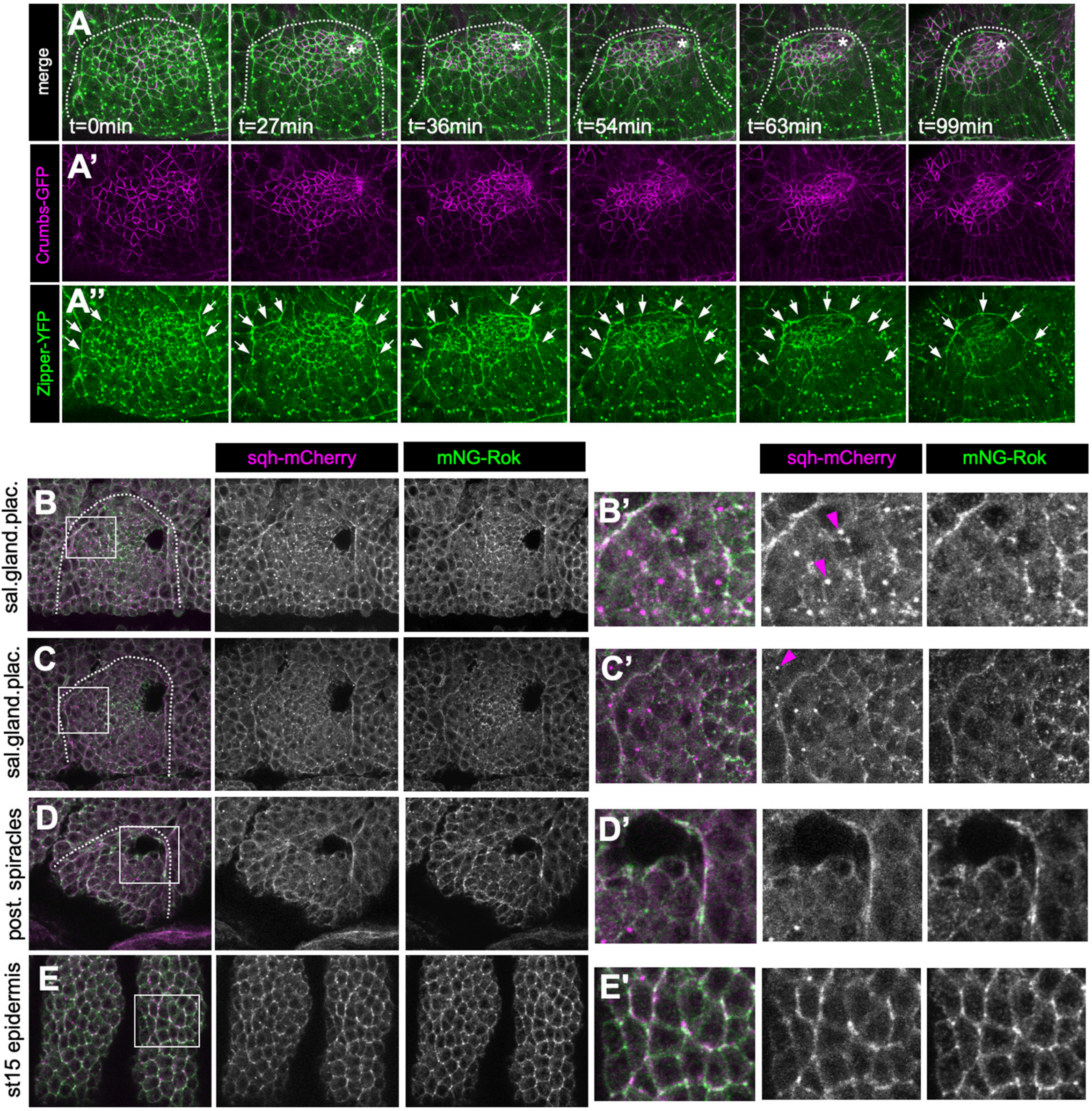


Sidor et al._Figure S1

***Figure S2, related to Figure 2. Crumbs membrane localisation locally affects Rok membrane localisation.***

Overexpression of Crumbs in stripes (white brackets and magenta arrows), using *en-Gal4 x UAS-Crumbs*, within the epidermis leads to spreading of Crumbs (magenta in **A-C**) to more basal positions along the lateral plasma membrane. This in turn leads to a basal displacement of mNG-Rok (green) along the lateral sides, visible through higher mNG-Rok membrane levels at a sub-apical position (**B',C'**, white arrows indicate mNG-Rok position) and loss of it at the apical-most lateral position (**A'**).

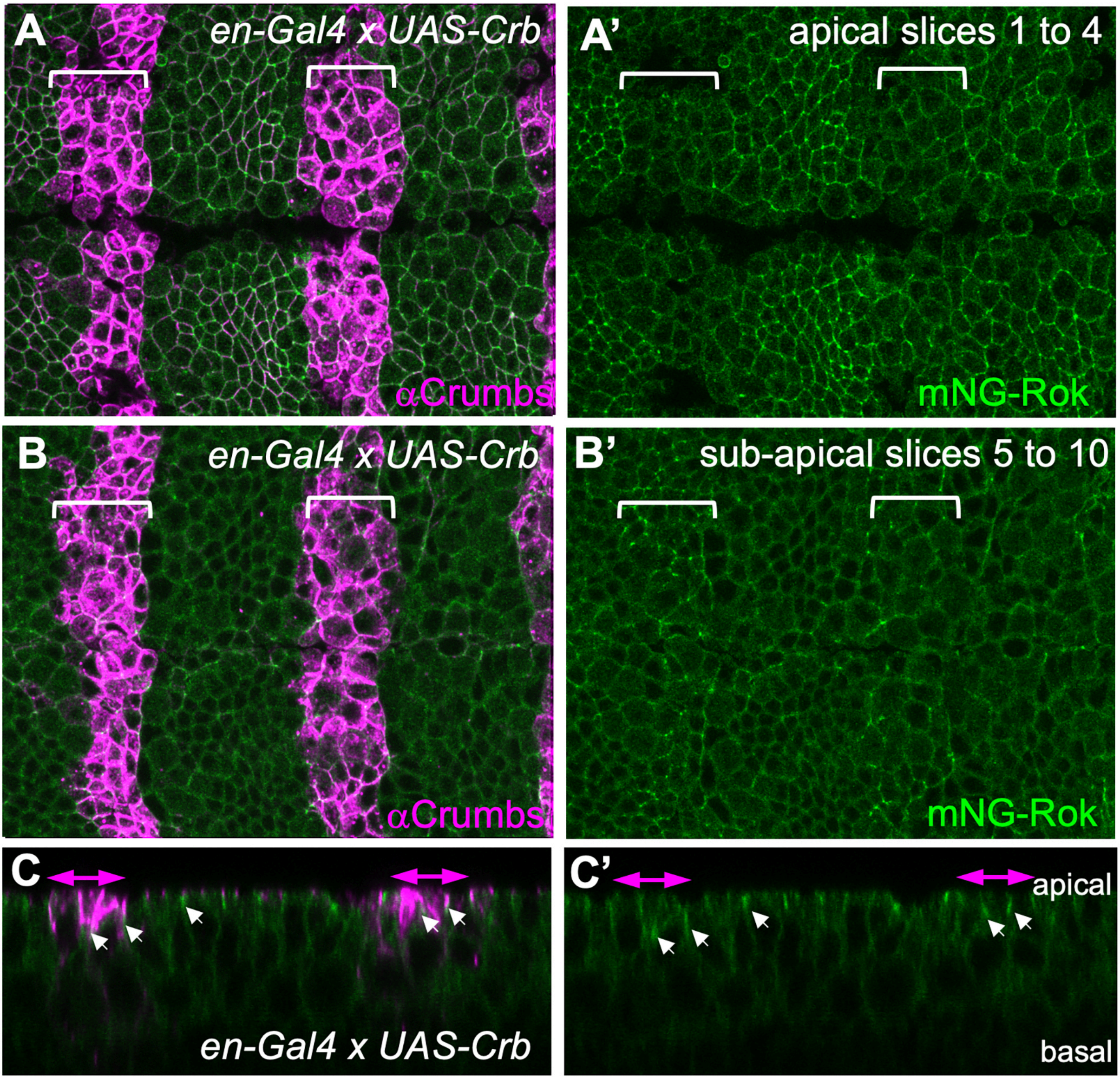


Sidor et al._ Figure S2

***Figure S3, related to Figure 3. FRAP analysis and in silico simulation of Rok dynamics at the placode boundary versus inside the placode.***

**A** Membrane versus cytoplasm enrichment of mNG-Rok in inner placodal cells (2.37) and surrounding epidermal cells (2.64). Data are represented as data points, mean and SEM. **B** Comparison of mNG-Rok levels in different membranes (membrane illustrated in the box), expressed as ratios of different membrane domains: boundary vs side (1.41), boundary vs inner (1.22), side vs inner (0.79), inner vs outer (1.59).

**C** Mobile fraction of mNG-Rok at placode boundary membranes (blue; value is 66%), side membranes (magenta; value is 92%) or inner membranes (orange; value is 77%), data points, mean and spread are shown. **D** Scatter plot of $k_{off}$ versus mobile fraction for boundary vs side and inner junctions, showing close clustering of side and inner and clear difference to boundary junctions.

**E** Outputs of *in silico* simulations as in Figure 3G, comparing the same combinations of $k_{off}$ values paired with different $k_{on}$ values (0.2; 0.3; 0.4). Color code for particle enrichment at junctions as in Figure 3G, numbers on representative junctions are the particle numbers derived from simulations.

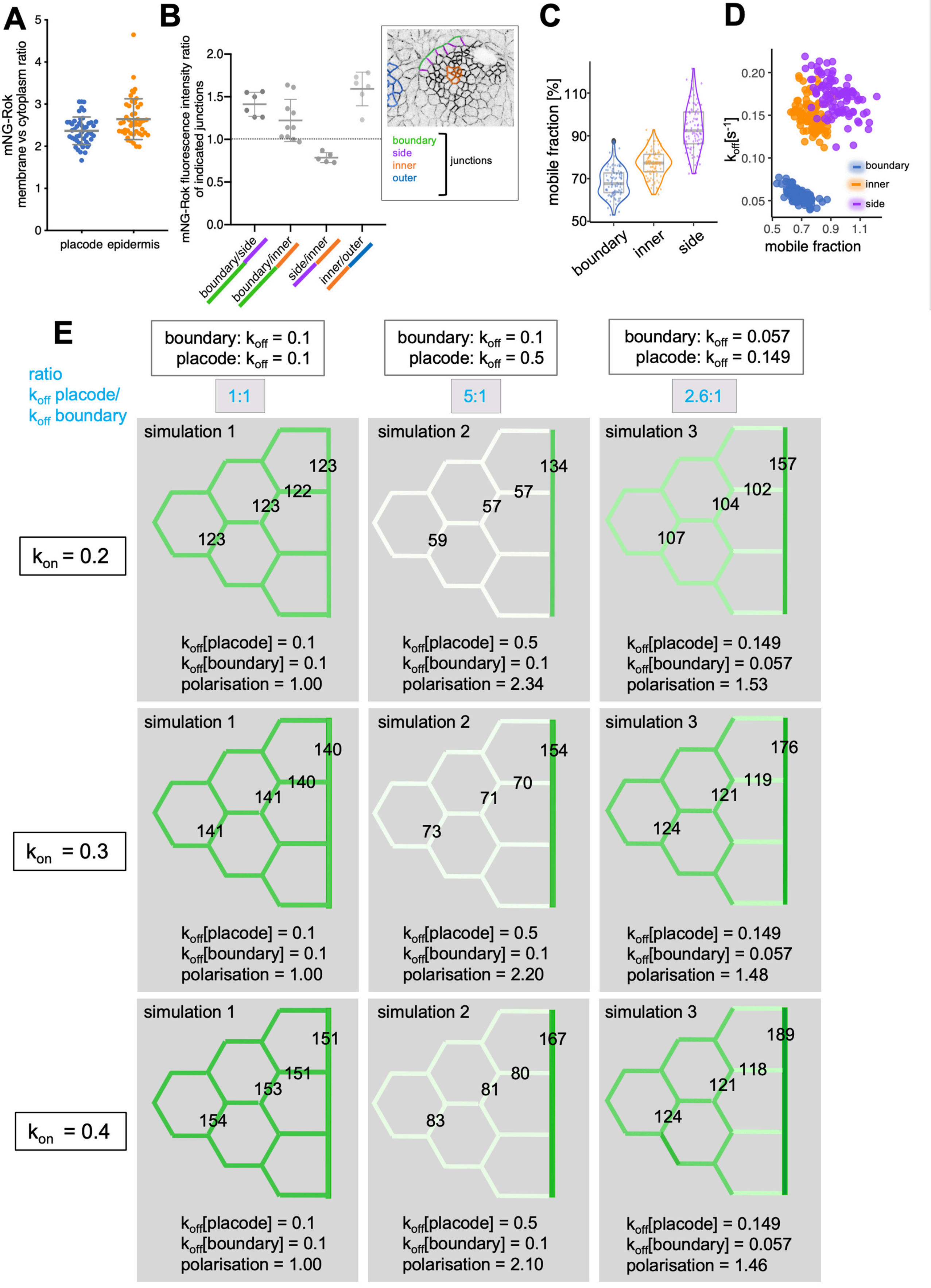


Sidor et al._Figure S3

***Figure S4, related to Figure 5. Analysis of predicted aPKC and Pak1 phosphorylation sites in Dm Rok.***

We used published predictive algorithms to identify potential aPKC and Pak1 phosphorylation sites in *Dm* Rok. Residues marked in yellow are predicted Pak1 sites (using DISPHOS) with a score >0.8, grey residues are predicted Pak1 sites (using DISPHOS) with a score between 0.6 and 0.8. Blue marks residues as predicted aPKC target sites (using NetPhos3.1). Green residues are predicted target sites for both aPKC and Pak1. Bold, underlined residues mark the RBD and PH domain. Conserved residues between human ROCK1 and *Dm* Rok that were found to be phosphorylated in EpH4 cells are shown in red and underlined (Ishiuchi and Takeichi, 2011).

### *Dm* Rho-kinase (isoform PA)

MPAGRETVTK QRSMDVERRR RANTLEREMR DPTSICNVDC LLDTVSALVS DCDHESLRRL
KNIEQYAAKY KPLAMQINQL RMNVEDFHFI KLIGAGAFGE VQLVRHKSSS QVYAMKRLSK
FEMMKRPDSA FFWEERHIMA HANSEWIVQL HFAFQDAKYL YMVMDFMPGG DIVSLMGDYD
IPEKWAIFYT MEVVLALDTI HNMGFVHRDV KPDNMLLDSY GHLKLADFGT CMRMGANGQV
VSSNAVGTPD YISPEVLQSQ GVDNEYGREC DWWSVGIFLY EMLFGETPFY ADSLVGTYGK
IMDHKNSLSF PPEVEISEQA KALIRAFLTD RTQRLGRYGI EDIKAHPFFR NDTWSFDNIR
ESVPPVVPEL SSDDDTRNFE DIERDEKPEE VFPVPKGFDG NHLPFIGFTY TGDYQLLSSD
TVDAESKEAN VANSGAASNN HGHGHNHRHR PSNSNELKRL EALLERERGR SEALEQQDAG
LRQQIELITK REAELQRIAS EYEKDLALRQ HNYKVAMQKV EQEIELRKKT EALLVETQRN
LENEQKTRAR DLNINDKVVS LEKQLLEMEQ SYKTETENTQ KLKKHNAELD FTVKSQEEKV
RDMVDMIDTL QKHKEELGQE NAELQALVVQ EKNLRSQLKE MHKEAENKMQ TLINDIERTM
CREQKAQEDN RALLEKISDL EKAHAGLDFE LKAAQGRYQQ EVKAHQETEK SRLVSREEAN
LQEVKALQSK LNEEKSARIK ADQHSQEKER QLSMLSVDYR QIQLRLQKLE GECRQESEKV
AALQSQLDQE HSKRNALLSE LSLHSSEVAH LRSRENQLQK ELSTQREAKR RFEEDLTQLK
STHHEALANN RELQAQLEAE QCFSRLYKTQ ANENREESAE RLSKIEDLEE ERVSLKHQVQ
VAVARADSEA LARSIAEETV ADLEKEKTIK ELELKDFVMK HRNEINAKEA ALATLKEAEN
**ELHKKLGQKA AEYEDLVQQH KKQQEELALM RSSKDEEITK LLDKCKNEVL LKQVAVNKLA**
**EVMNRR**DSDL PKQKNKARST AELRKKEKEM RRLQQELSQE RDKFNQLLLK HQDLQQLCAE
EQQLKQKMVM EIDCKATEIE NLQSKLNETA SLSSADNDPE DSQHSSLLSL TQD**SVFEGWL**
**SVPNKQNRRR GHGWKRQYVI VSSRKIIFYN SDIDKHNTTD AVLILDLSKV YHVRSVTQGD**
**VIRADAKEIP RIFQLLYAGE GASHRPDEQS QLDVSVLHGN CNEERPGTIV H**KGHEFVHIT
YHMPTACEVC PKPLWHMFKP PAAYECKRCR NKIHKEHVDK HDPLAPCKLN HDPRSARDML
LLAATPEDQS LWVARLLKRI QKSGYKAASY NNNSTDGSKI SPSQSTRSSY KPYAVNVQRS
ATLPANSSLK

→ predicted Pak1 sites score>0.8 (DISPHOS results)
→ predicted Pak1 sites 0.6<score<0.8 (DISPHOS results)
→ predicted aPKC sites (NetPhos3.1)
→ putative sites for both aPKC and Pak1
→ peptides tested in vitro
→ conserved residues between human and *Drosophila* that are phosphorylated in EpH4 cells
→ **bold and underlined** residues mark the RBD and PH domain

Sidor et al._Figure S4

***Figure S5, related to Figure 7. mNG-Rok dynamics under Pak1-level modulation.***

**A** Mobile fraction of mNG-Rok at placode boundary membranes and inner membranes when Pak1 levels are modulated: *UAS-Pak1*$^{myr}$/boundary (dark blue; value is 87%), *UAS-Pak1*$^{myr}$/inner(orange; value is 71%), *pak1*$^{14}$/boundary (light blue; value is 58%), *pak1*$^{14}$/inner (light brown; value is 70%). Data points, mean and spread are shown. **B** Scatter plot of $k_{off}$ versus mobile fraction for boundary vs inner junctions when Pak1 levels are modulated, showing close clustering of *UAS-Pak1*$^{myr}$ for both boundary and inner junctions, as well as the overlap for the *pak1*$^{14}$ mutant for both boundary and inner junctions.

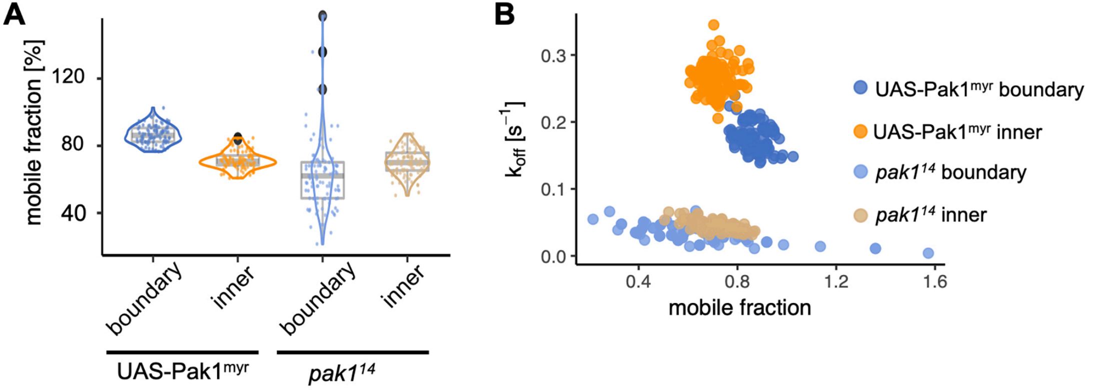


Sidor et al._Figure S5
