# Supplementary material for: Rho-Kinase Planar Polarization at Tissue Boundaries Depends on Phospho-regulation of Membrane Residence Time
Source: Dev Cell. 2020 Feb 10;52(3):364–378.e7. doi: 10.1016/j.devcel.2019.12.003 (PMC7008249; doi:10.1016/j.devcel.2019.12.003)
Supplement: Document S2. Article plus Supplemental Information [file mmc5.pdf]

Developmental Cell

Article

Rho-Kinase Planar Polarization at Tissue Boundaries Depends on Phospho-regulation of Membrane Residence Time

Graphical Abstract

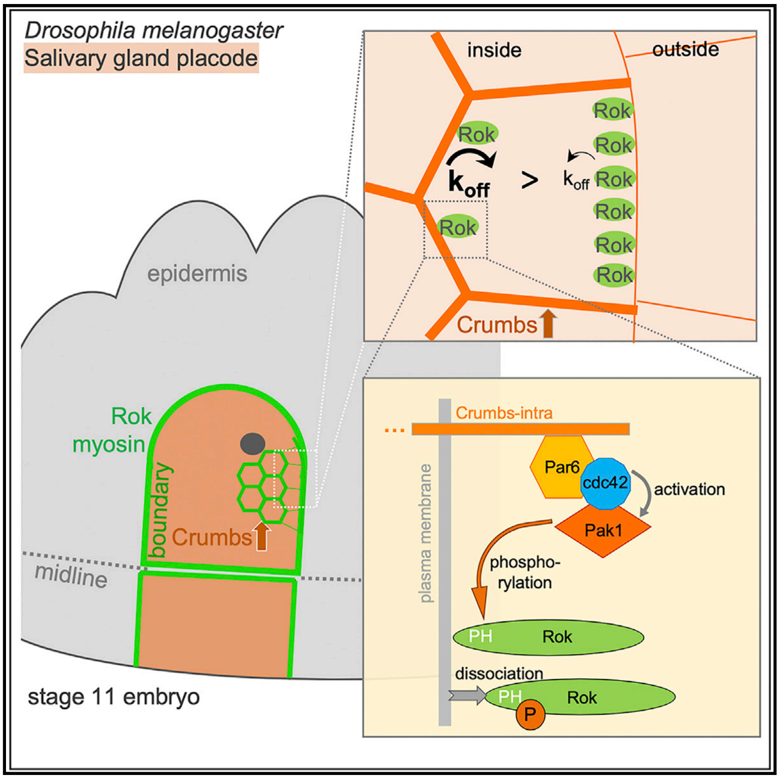

Highlights

- Rho-kinase is planar polarized at tissue boundaries, complementary to Crumbs
- Crumbs and downstream Pak1 modulate Rok residence time by affecting k_{off}
- Pak1 can phosphorylate Rok near the PH and Rho-binding domains
- Rok phosphorylation affects residence time and allows polarization at boundaries

Authors

Clara Sidor, Tim J. Stevens, Li Jin, Jérôme Boulanger, Katja Röper

Correspondence

clara.sidor@univ-amu.fr (C.S.), kroeper@mrc-lmb.cam.ac.uk (K.R.)

In Brief

Sidor et al. show that a modulation of Rok's k_{off} through phosphorylation of its membrane association regions by Pak1 downstream of Crumbs can drive planar polarization of Rok at tissue boundaries.

Sidor et al., 2020, Developmental Cell 52, 364–378
February 10, 2020 © 2019 MRC Laboratory of Molecular Biology.
Published by Elsevier Inc.
https://doi.org/10.1016/j.devcel.2019.12.003

Developmental Cell
Article

Rho-Kinase Planar Polarization at Tissue Boundaries Depends on Phospho-regulation of Membrane Residence Time

Clara Sidor,[1,2,*] Tim J. Stevens,[1] Li Jin,[1] Jérôme Boulanger,[1] and Katja Röper[1,3,**]
[1]MRC Laboratory of Molecular Biology, Francis Crick Avenue, Cambridge Biomedical Campus, Cambridge, UK
[2]Present address: IBDM-Institut de Biologie du Développement de Marseille (UMR 7288), CNRS & Aix-Marseille Université, Case 907, Parc Scientifique de Luminy, 13288 Marseille Cedex 9, France
[3]Lead Contact
*Correspondence: clara.sidor@univ-amu.fr (C.S.), kroeper@mrc-lmb.cam.ac.uk (K.R.)
https://doi.org/10.1016/j.devcel.2019.12.003

SUMMARY

The myosin II activator Rho-kinase (Rok) is planar polarized at the tissue boundary of the *Drosophila* embryonic salivary gland placode through a negative regulation by the apical polarity protein Crumbs that is anisotropically localized at the boundary. However, in inner cells of the placode, both Crumbs and Rok are isotropically enriched at junctions. We propose that modulation of Rok membrane residence time by Crumbs' downstream effectors can reconcile both behaviors. Using FRAP combined with *in silico* simulations, we find that the lower membrane dissociation rate (k_{off}) of Rok at the tissue boundary with low Crumbs explains this boundary-specific effect. The S/T kinase Pak1, recruited by Crumbs and Cdc42, negatively affects Rok membrane association *in vivo* and *in vitro* can phosphorylate Rok near the pleckstrin homology (PH) domain that mediates membrane association. These data reveal an important mechanism of the modulation of Rok membrane residence time via affecting the k_{off} that may be widely employed during tissue morphogenesis.

INTRODUCTION

Tissues arise during development through specification of primordia that will then initiate morphogenetic movements (Castelli-Gair Hombria and Bovolenta, 2016). Many primordia are epithelial in nature and give rise to tubular epithelial organs, such as the lung, kidney, and intestine in vertebrates or the equivalent organs in invertebrates. How are epithelial primordia physically set aside from the surrounding tissue, apart from the inductive change in transcription factor expression? We know from studies in the *Drosophila* early embryonic epidermis as well as in larval wing discs that differently fated compartments are physically clearly segregated, and cell mixing across compartment boundaries is restricted (Dahmann and Basler, 1999; Tepass et al., 2002). In both tissues, this seems to be in part achieved through an increased tension at the compartment boundary that coincides with junctional accumulation of actomyosin into a seemingly supracellular structure, a so-called actomyosin cable (Röper, 2013). In the embryonic epidermis, the actomyosin cables found at parasegmental boundaries physically restrain boundary-challenging divisions within the correct compartment (Monier et al., 2010). The molecular mechanisms that drive actomyosin cable assembly are not well understood, and where aspects have been uncovered, a variety of tissue-specific mechanisms seem to contribute. Parasegmental cables, for instance, arise over time from dorsoventrally polarized junctional myosin accumulations (Tetley et al., 2016) that might themselves depend on a code of Toll receptor expression within the early epidermis (Paré et al., 2014). In the wing disc, the dorsoventral boundary requires Notch-signaling (Major and Irvine, 2005, 2006). Actomyosin-based compartment boundaries are not restricted to invertebrates but have in fact also been found in vertebrates, with key examples being the rhombomere boundaries in the mammalian hindbrain (Calzolari et al., 2014) as well as the neural plate-ectoderm boundary during neurulation (Galea et al., 2017).

We have previously identified that an actomyosin cable is positioned at the boundary of the salivary gland placode in the *Drosophila* embryo (Röper, 2012). Two epithelial placodes of about 100 cells on either side of the ventral midline become specified at stage 10 of embryogenesis and will invaginate to form the two salivary glands (Girdler and Röper, 2014; Sidor and Röper, 2016) (Figures 1A and 1A′). This invagination process is driven by a combination of isotropic apical constriction, mediated by dynamic pools of apical-medial actomyosin, and directed cell intercalation, mediated by apical junctional pools of actomyosin (Booth et al., 2014; Sanchez-Corrales et al., 2018). The placodes are contained within parasegment 2 of the embryo, and remnants of previous parasegmental actomyosin cables are specifically retained near the ventral portion of the placode, while a new section forms around the dorsal part, so that by mid stage 11 a circumferential actomyosin cable surrounds each placode (Röper, 2012). The formation of this cable is transcriptionally initiated, as it is lacking in mutants for the most upstream specifying transcription factor Sex combs reduced (Henderson and Andrew, 2000; Röper, 2012). We have previously identified the apical transmembrane protein Crumbs as a key determinant of actomyosin cable positioning at the boundary of the placode. Crumbs levels are strongly increased within the placode, whereas levels

 © 2019 MRC Laboratory of Molecular Biology. Published by Elsevier Inc.
This is an open access article under the CC BY license (http://creativecommons.org/licenses/by/4.0/).

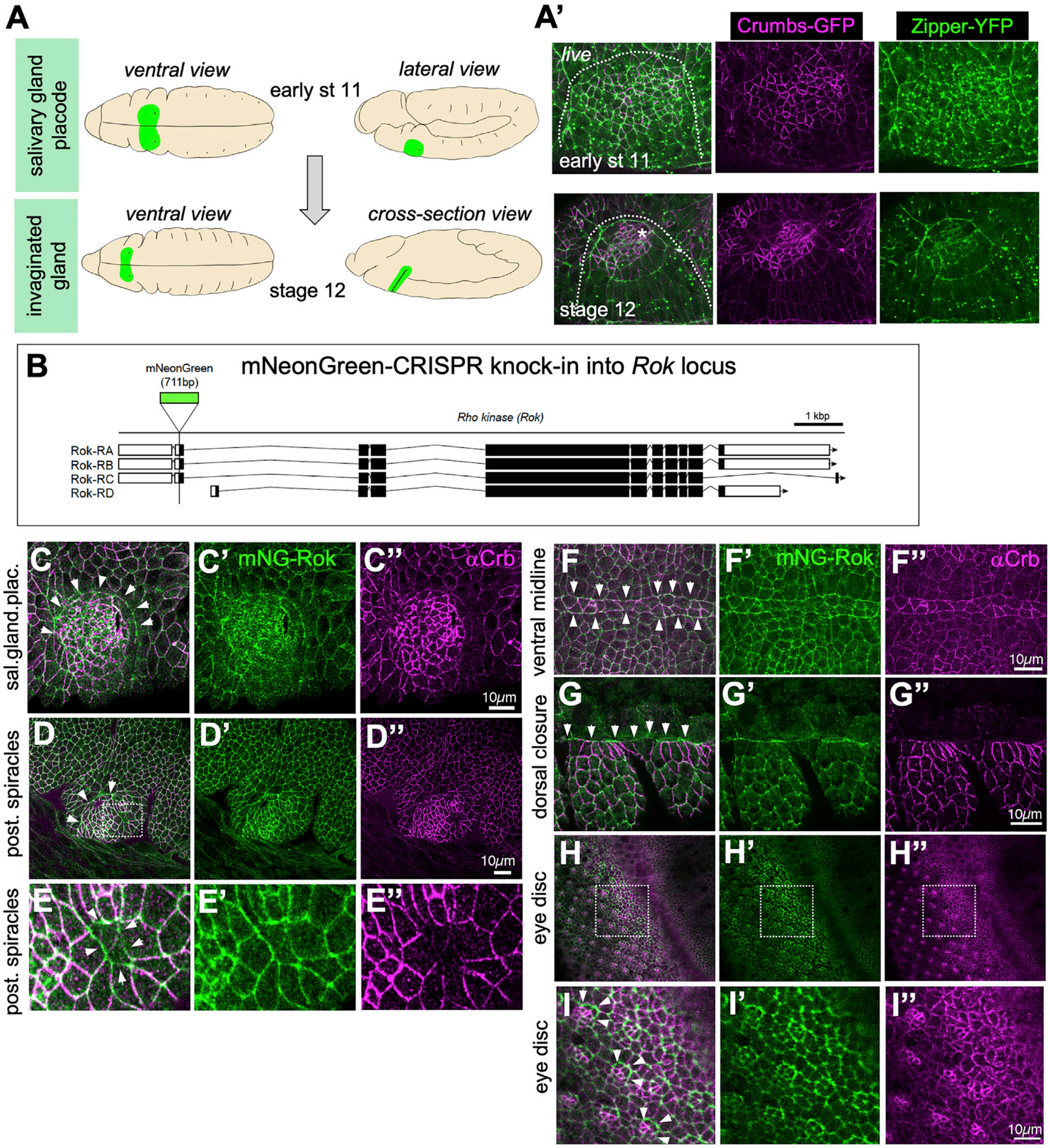

Figure 1. Widespread Apical Planar Polarization of Crumbs and Rho-Kinase during Morphogenesis

(A) The salivary gland develops from an epithelial placode on the ventral side of the embryo (green area). Cells become specified at late stage 10/early stage 11, and by stage 12 most secretory cells have invaginated to form a tube inside the embryo.

(A′) The salivary gland placode boundary shows a strong enrichment of myosin II (*Zipper-YFP*, green) into a supracellular cable, complementary to anisotropic enrichment of Crumbs (*Crumbs-GFP*, magenta) in the placodal boundary cells (Röper, 2012); stills of a time-lapse video of matching stages to schematics in (A). See also Figure S1 and Video S1.

(B) Genomic locus of *Drosophila* Rok, indicating exon usage in splice variants and the position of the mNeon-Green (mNG) tag inserted at the N terminus.

(C–I) mNG-Rok enrichment (green) complementary to Crumbs anisotropic localization (magenta) at many epithelial boundaries: in the embryo, the salivary gland placode (C–C″), boundary of the posterior spiracle placode (D–D″), spiracular hair precursors (E and E′), ventral midline (F–F″), leading edge during dorsal closure (G–G″), as well as the boundaries of specified photoreceptor clusters during larval eye disc morphogenesis (H–I″). Boxes in (D) and (H) indicate magnifications in (E)–(E″) and (I)–(I″), respectively. Arrowheads point to the boundaries in Crumbs levels that show strong mNG-Rok accumulation.

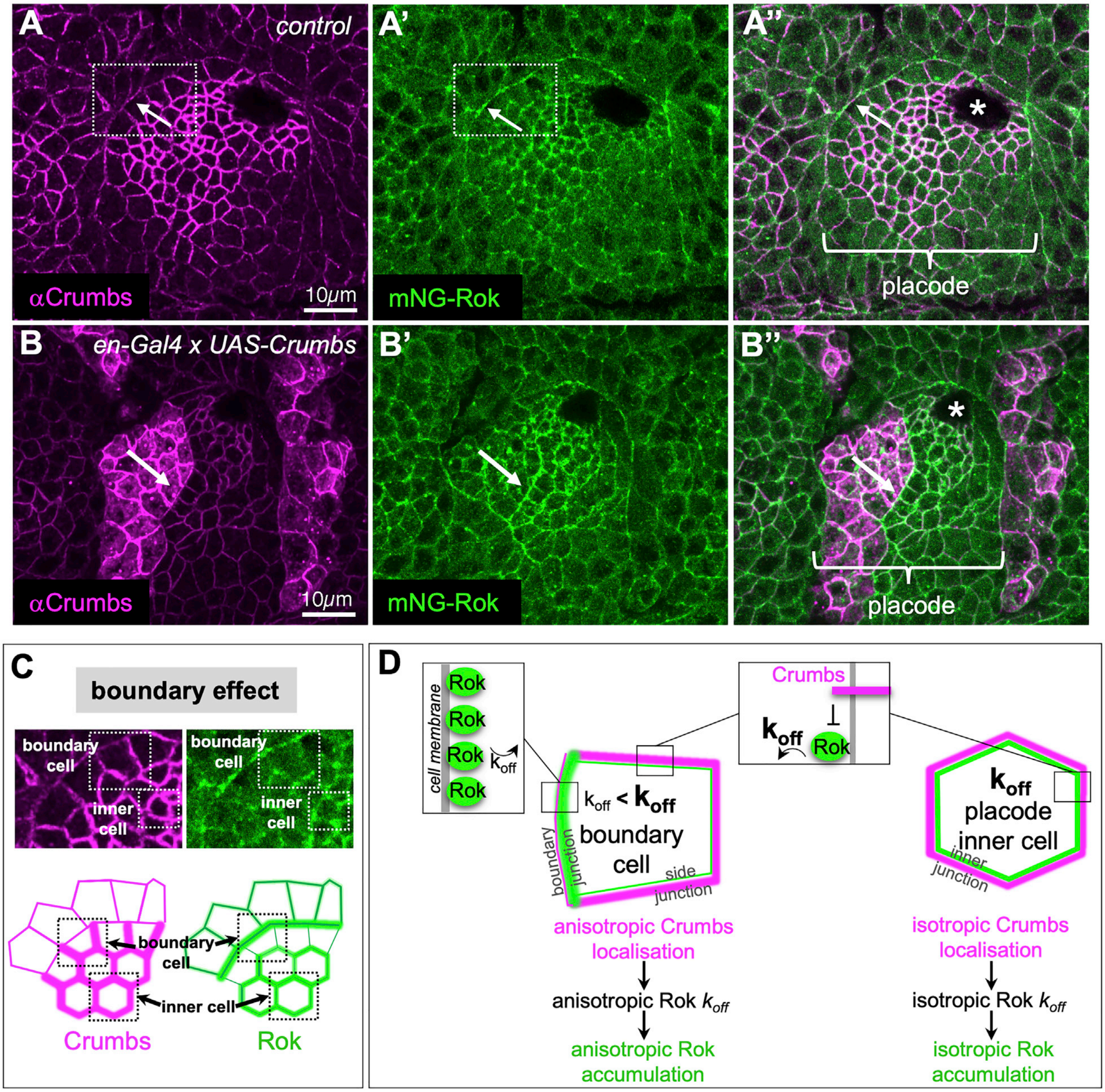

Figure 2. Rho-Kinase Planar Polarization at the Boundary of the Salivary Gland Placode

(A–A″) Crumbs is highly enriched in the salivary gland placode and reduced in levels in the surrounding epidermis (A), leading to its strong anisotropic localization in boundary cells due to homophilic interactions (white arrows in A and A″). mNG-Rok is enriched complementary to Crumbs in boundary cells but also strongly enriched isotropically in junctions in the inner cells of the placode (A′, green).

(B and B′) Introduction of a new boundary of Crumbs protein levels within the placode, using *en-Gal4* x *UAS-Crumbs* (B, magenta) leads to accumulation of mNG-Rok at the new boundary (B′, green), complementary to anisotropic Crumbs at the new boundary. See also Figure S2.

(C) The anisotropic and complementary localization of Crumbs and mNG-Rok shows a "boundary effect," the negative regulatory effect that Crumbs exerts on Rok localization is only apparent in cells that show Crumbs anisotropy (boundary cell), whereas isotropic Crumbs accumulation in the center of the placode (inner cell) does not prevent high levels of junctional Rok accumulation.

(D) A dynamic model of the modulation of Rok residence time at the membrane, depending on local levels of Crumbs, could explain planar polarization of Rok in cells showing Crumbs anisotropy while preserving isotropic Rok localization in cells with isotropic Crumbs. Asterisks in (A″) and (B″) indicate the invagination point.

are reduced in the epidermal cells surrounding the placode (Figures 1C″ and 2A), and the placodal increase is transcriptionally regulated (Kerman et al., 2008; Myat and Andrew, 2002). Crumbs' ability to form homophilic interactions (Fletcher et al., 2012; Röper, 2012; Zou et al., 2012) leads to a highly anisotropic distribution of Crumbs apically within placodal cells at the placode

boundary: this Crumbs anisotropy leads to accumulation of actomyosin and ectopic Rho-kinase (Rok) reporters at the placode boundary, away from membranes with high levels of Crumbs. Moreover, ectopic boundaries of high versus low Crumbs levels lead to ectopic myosin cable formation (Röper, 2012). Thus, Crumbs exerts a negative regulatory effect on actomyosin cable formation, likely through the upstream myosin activator Rok. Based on the studies in mammalian cells that reported that the kinase aPKC is able to phosphorylate and thereby inactivate Rok (Ishiuchi and Takeichi, 2011), we previously suggested that the effect in the placode was mediated by aPKC, which in fly embryos closely follows Crumbs distribution (Röper, 2012). Complementary localization of Crumbs and actomyosin is not restricted to flies but has also more recently been reported in mouse (Ramkumar et al., 2016).

The negative influence of Crumbs on Rok localization and activity is clear at the placode boundary where Crumbs anisotropy triggers the actomyosin cable assembly in boundary junctions with lower levels of Crumbs. However, in the inner placodal cells, where Crumbs is high because of transcriptional control and Rok is required for the actomyosin-dependent invagination process, both Crumbs and Rok membrane levels are high and isotropic. Thus, it appears that it is not the overall levels of Crumbs that negatively affect Rok but rather the difference in levels experienced within a single cell at the boundary that allows negative regulation of Rok. This is also supported by the fact that introduction of a new step change in Crumbs levels within the placode, now adding a stripe of Crumbs expression even higher than the already elevated placodal levels, triggers Crumbs anisotropy and ectopic myosin cable formation (Röper, 2012). Here, we propose a molecular mechanism by which these different scenarios can be reconciled, based on a modulation of Rok residence time downstream of Crumbs at the apical junctional plasma membrane, mediated by modulation of the k_{off} through phosphorylation of Rok. We suggest that such a mechanism might be widely used in membrane receptors to allow a combination of patterning activity with other molecular functions.

RESULTS

Rok Is Planar Polarized and Complementary to Crumbs at the Placode and Other Tissue Boundaries

We previously proposed that the apical transmembrane protein Crumbs actively influences myosin II accumulation through a negative regulatory effect on Rok, the most common activator of myosin II during morphogenetic processes (Röper, 2012). However, current visualization of Rok localization and activity depends on overexpression of tagged active or kinase-dead versions of the protein (Simões et al., 2010). In order to examine endogenous Rok localization, we used CRISPR/Cas9 to engineer an endogenously N-terminally tagged Rok with the bright mNeon-Green (mNG) fluorescent protein, tagging major isoforms A, B, and C (Shaner et al., 2013) (Figure 1B). Isoform D has an alternative start, but it is not expressed during embryogenesis (modENCODE Consortium et al., 2010). Importantly, mNG-Rok did not form aggregates similar to many of the overexpression lines (see below) and mNG-Rok flies were homozygous viable, indicating that Rok function was not impaired by the mNG-tag.

In epithelial cells of the epidermis, as well as in larval imaginal discs, endogenous mNG-Rok localized similarly to previously described tagged Rok, being enriched both in the apical junctional as well as in the apical-medial region of certain cells (Figures 1C–1H). At several boundaries of differently fated epidermal domains, such as in the embryo, the salivary gland placode (Figure 1C), posterior spiracles (Figures 1D and 1E), ventral midline, (Figure 1F), epidermal leading edge/amnioserosa interface (Figure 1G), and larval eye disc (Figures 1H and 1I), mNG-Rok was strongly enriched at the boundary, as was the downstream morphogenetic effector myosin II (Figure S1) (Jacinto et al., 2002; Röper, 2012). These were also all boundaries where Crumbs levels show a clear step change of high versus low expression.

Crumbs Induces Rok Planar Polarization at the Placode Boundary

In the salivary gland placode, mNG-Rok was enriched apically in junctions in comparison to the surrounding epithelium (Figures 1C′ and 2A–2A″). However, in placode boundary cells, where the Crumbs protein is highly anisotropically localized within the sub-apical domain (Figure 2A, arrow, and 2C), apical junctional Rok was planar polarized. In these boundary cells, mNG-Rok accumulated in junctions with lower levels of Crumbs, the boundary junctions, and appeared depleted from junctions with high Crumbs levels, the side junctions (Figures 2A–2A″ and 2C), suggesting that Crumbs negatively regulates endogenous Rok membrane accumulation. In order to examine the effect of Crumbs on endogenous Rok membrane localization, we induced Crumbs overexpression in a stripe of cells in mNG-Rok embryos using the *en-Gal4* driver (using the UAS-Gal4 system) (Brand and Perrimon, 1993). This ectopic stripe of higher Crumbs levels created a new high/low boundary of the Crumbs protein within the salivary gland placode (Figure 2B, arrow). At the new boundary, Crumbs localization was highly anisotropic, with Crumbs enriched in junctions with neighboring cells that also expressed high levels of Crumbs and lower at junctions with the surrounding placode cells (Figure 2B). Again, only within the cells at the stripe boundary, where Crumbs was highly anisotropic, mNG-Rok was planar polarized and enriched at the new ectopic boundary junctions with lower levels of Crumbs, and it was depleted from junctions with higher levels of Crumbs (Figures 2B′ and B″). This negative regulatory effect was also visible within the apical-basal extent of the epidermal cells, as an expansion of the apico-lateral distribution of Crumbs due to ectopic overexpression leads to a basal shift in mNG-Rok localization within the lateral membrane (Figure S2). Thus, Crumbs negatively regulates endogenous Rok membrane association.

A Model to Explain Rok Polarization at the Boundary via Modulation of Rok Dynamics

We were intrigued by the fact that the negative regulatory effect of Crumbs appeared to only affect Rok in cells at the boundary of the salivary gland placode but not all throughout the tissue. In inner placodal cells, where Crumbs was highly enriched isotropically at all sub-apical junctions (due to transcriptional upregulation), mNG-Rok was able to localize at apical junctions despite the high Crumbs levels (Figures 2A–2A″ and 2C). In contrast, in boundary cells where Crumbs localization was highly

anisotropic, mNG-Rok was planar polarized. Furthermore, an ectopic Crumbs boundary with much higher levels of Crumbs present could also polarize Rok (Figures 2B–2B″). Thus, it was the anisotropic distribution of Crumbs within a cell, rather than its absolute levels in the plasma membrane, that affected Rok membrane localization (Figure 2C).

One way to mechanistically reconcile these two situations (boundary versus inner placode) is to consider the dynamics of Rok membrane localization (Figure 2D). If we assume that at equilibrium Rok is able to associate and dissociate from the cell membrane at specific rates, Crumbs could influence Rok membrane accumulation by selectively increasing the Rok membrane dissociation rate (k_{off}). We propose that within inner placodal cells, high levels of Crumbs in all apical junctions would lead to a higher, though isotropic, Rok turnover. With Rok membrane recruitment, i.e., k_{on}, remaining unaffected (in inner cells just as in boundary cells), Rok would still localize at the plasma membrane of these inner cells. Within the boundary cells, Rok would dissociate more often from junctions with high levels of Crumbs (side junctions) and would therefore accumulate at junctions with lower levels of Crumbs and a lower k_{off} (boundary junction), resulting in the planar polarization of Rok at this tissue boundary.

Endogenous Rok k_{off} Is Lower at the Boundary of the Placode and Leads to Planar Polarization of Rok in Simulations

The above model clearly predicts that Rok's mobility and in particular Rok's k_{off} at the boundary junctions should differ from Rok's k_{off} at other junctions within the placode, both the side junctions of a boundary cell as well as inner junctions of a placode inner cell (with both types of junctions showing the same high levels of Crumbs). To test this prediction, we performed fluorescence recovery after photobleaching (FRAP) experiments in stage 11–12 embryos expressing the endogenously tagged mNG-Rok (Figures 3A and 3B). We bleached small circular regions of mNG-Rok at the apico-lateral plasma membrane and imaged at ~500–700-ms intervals to capture the mNG-Rok fluorescence intensity pre- and post-bleach. Supporting our predictions, mNG-Rok located at junctions within the placode, both inner junctions within the center of the placode as well as side junctions of boundary cells, recovered significantly faster than mNG-Rok located at the boundary (Figures 3B–3D). As demonstrated in the Supplemental Information, given our experimental settings, the recovery rate can be interpreted as a k_{off} of a reaction-diffusion equation (Bulinski et al., 2001; Sprague et al., 2004). k_{off} values were estimated from fluorescence recovery as 0.148 (±0.018) s^{-1} for the inner placodal cells, 0.173 (±0.024) s^{-1} for side membranes of boundary cells, and 0.057 (±0.008) s^{-1} for the boundary membrane of boundary cells. Estimated k_{off} values were found to be significantly lower in boundary junctions compared with junctions within the placode, both side and inner junctions (p values [bootstrap/boxplot], 0.0002; Figures 3C and 3D). Thus, the difference in the dynamic behavior of mNG-Rok at junctions forming the boundary of the placode compared with other junctions within the placode supports the model for Rok planar polarization presented above.

In order to assess whether the difference in Rok's k_{off} measured at the placode boundary and within the side and inner junctions of the placode was in itself sufficient to produce planar polarization of Rok at the tissue boundary, we developed an *in silico* simulation of the process, using particle-based stochastic reaction diffusion (Figures 3E, 3F, and S3; for details of the implementation, see STAR Methods). We tested a variety of k_{off} combinations for the boundary and side and inner placodal cell membranes (keeping the k_{on} constant, Figure S3). Uniform k_{off} values in all membranes led, as expected, to isotropic membrane accumulation of Rok (Figure 3G, simulation 1). Setting a high k_{off} in inner junctions compared with boundary junctions led to a strong Rok polarization at the boundary but markedly reduced Rok membrane localization in inner cells (Figure 3G, simulation 2). By contrast, k_{off} values deduced from the *in vivo* FRAP measurements of mNG-Rok were sufficient to trigger Rok planar polarization in simulated boundary cells, at a level similar to the polarization measured *in vivo* (*in silico* polarization value of 1.48, Figure 3G, simulation 3; compare with values for mNG-Rok *in vivo* in Figures 6E and 7G below). Under these conditions, Rok accumulation at the membrane of inner cells with a higher k_{off} was preserved, leading to a pattern that closely resembled Rok localization in the salivary gland placode *in vivo* (Figures 1C′ and 2A′).

Thus, modulation of the Rok membrane k_{off} within the placode is sufficient to elicit Rok planar polarization specifically at the tissue boundary while preserving Rok membrane accumulation in the rest of the tissue. We next investigated the molecular basis for this mechanism.

The Rok C-Terminal Region Is Required for Its Planar Polarization

Drosophila Rok is a large protein of 1,391 amino acids, containing an N-terminal kinase domain followed by a coiled-coil region and a C-terminal Shroom-binding domain (SBD), Rho-binding domain (RBD), and PH domain (PH) (Figure 4A) (Amano et al., 2010; Simões et al., 2014). In order to identify which of these domains were required for apical junctional planar polarization, truncated versions of a Venus-tagged kinase-dead Rok localization reporter were overexpressed in embryos using the UAS-Gal4 system under the control of *Daughterless-Gal4*, an early ubiquitous zygotic driver (Simões et al., 2014).

The Venus-Rok[K116A]-ΔSBD lacking the coiled coil and SBD was able to accumulate in apical membranes (at lower levels than the control Venus-Rok[K116A]) and could still polarize at the placode boundary (Figure 4B versus Figures 4C and 4F′). Venus-Rok constructs lacking the RBD, Venus-Rok[K116A] ΔRBD, or the PH, Venus-Rok[K116A]ΔPH, although showing a strongly reduced overall membrane accumulation, were able to localize at low levels to the membrane. Both constructs failed to polarize at the tissue boundary (Figures 4D–4E′ and 4F′). Thus, in addition to promoting Rok apical junctional membrane recruitment, both the RBD and the PH are important for Rok planar polarization in boundary cells.

Rok membrane recruitment has been shown to be dependent on the small GTPase Rho and its exchange factor RhoGEF2 (Amano et al., 2010; Mason et al., 2016; Nakamura et al., 2017). In order to assess whether Rok planar polarization is caused by planar polarization of these upstream activators, we examined the localization of a superfolderGFP (sfGFP)-tagged version of RhoGEF2 (Sarov et al., 2016) and the GFP-tagged RBD of Anillin (AniRBD-GFP), a reporter for activated GTP-

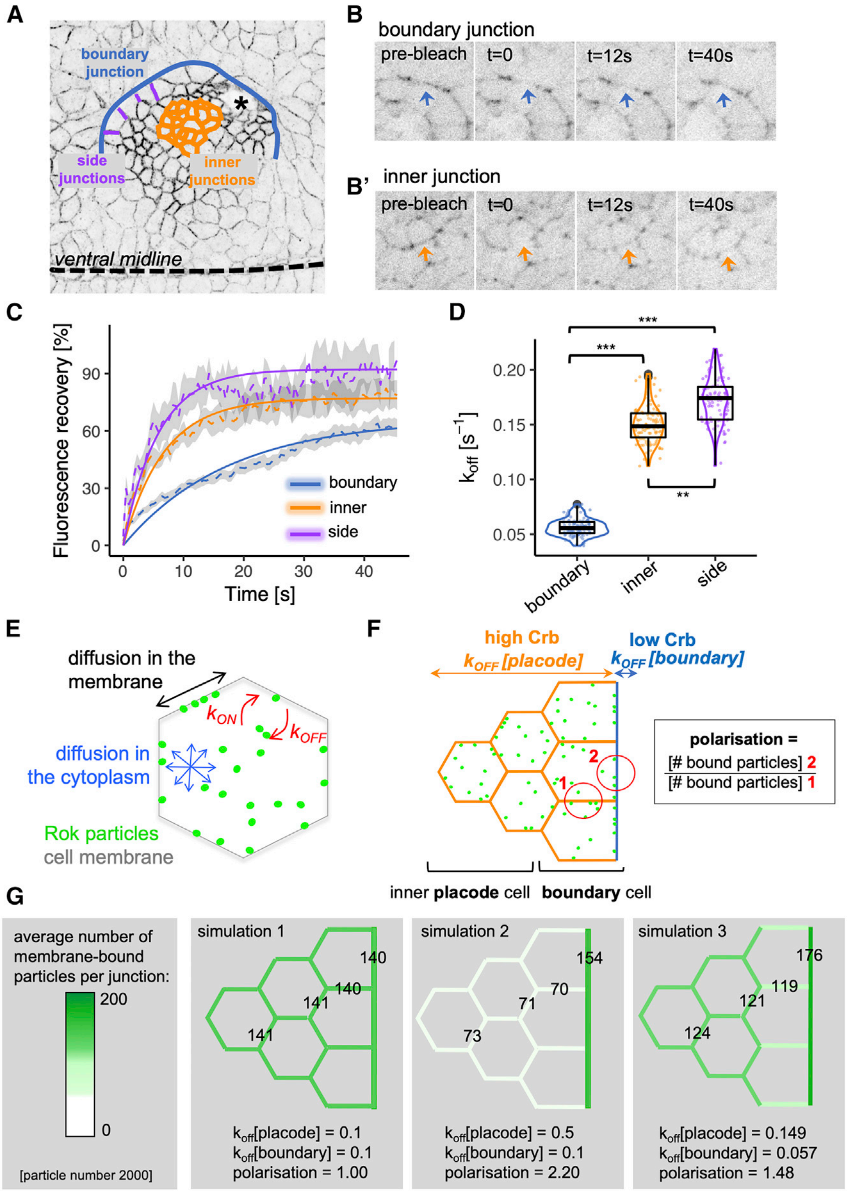

Figure 3. FRAP Analysis and *In Silico* Simulation of Rok Dynamics at the Boundary versus inside the Placode

(A) Schematic of the placode indicating the boundary with low levels of Crumbs (blue), the side membranes of boundary cells with high levels of Crumbs (magenta), and the inner placodal cells with isotropically high levels of Crumbs (orange) where FRAP analysis was performed. The invagination point (asterisk) and ventral midline position are indicated.

(B and B′) Examples of boundary (B) and inner (B′) cell junctions of mNG-Rok embryos during FRAP analysis, arrows indicate the positions of the bleached regions (bleach at t = 0). See also Videos S2 and S3.

(C and D) Recovery curves fitted to data of FRAP experiments for boundary junctions (blue, n = 27), side junctions (magenta, n = 7), and inner cell junctions (orange, n = 13) (C). k_{off} values were estimated from the fluorescence recovery for the boundary as 0.057 (±0.008), inner placodal membranes as 0.149 (±0.018), and side membranes as 0.173 (±0.024), and inner and side membranes were found to be significantly different from the boundary using a bootstrap procedure, with the p values determined as 0.0002 for both (***) (D). Data represented are bootstrap sample, median, and quartiles.

(E) We modeled the Rok planar polarization *in silico* using particle-based stochastic reaction diffusion.

(F) A group of cells representing boundary and inner placodal cells are modeled, imposing different k_{off} values for the boundary with low Crumbs levels (region 2, blue) and a higher k_{off} for membranes with high levels of Crumbs, such as side membrane and inner placodal membranes (region 1, orange). (G) Examples of steady-state outputs of the simulation, with simulation 1 depicting no difference in k_{off}, simulation 2 assuming a 5-fold difference in k_{off}, and simulation 3 using the k_{off} values determined by FRAP as the input; numbers on representative junctions are the particle numbers derived from simulations. See also Figure S3.

bound Rho (Munjal et al., 2015). Neither RhoGEF2-sfGFP nor AniRBD-GFP showed any apical junctional planar polarization within the boundary cells of the salivary gland placode (Figures 4G–4I). Thus, Rok planar polarization is not driven by an upstream polarization of active Rho.

These data suggest that, although Rho binding is important for Rok membrane recruitment, once at the membrane, Rok planar polarization is regulated by a Rho-independent mechanism involving the Rok C-terminal membrane association region.

Phosphorylation of the Rok C-Terminal Region by Pak1 Regulates Rok Membrane Association

Alternatively, regulation of Rok membrane association could be achieved via modulation of Rok's binding to Rho or to phospholipids, for instance, through phosphorylation in the vicinity of Rok's RBD or PH. Phosphorylation near phospholipid-interacting sequences in several Par-complex substrates has recently been suggested to inhibit their membrane binding (Bailey and Prehoda, 2015). We had previously suggested that Crumbs could negatively regulate Rok through one of its downstream interactors, aPKC (Figure 5A) (Röper, 2012), which binds to the Crumbs intracellular domain through its binding partner Par6 (Bulgakova and Knust, 2009). Previous data from mammalian tissue culture cells revealed that aPKC could phosphorylate human ROCK1, one of the two mammalian Roks, near the RBD and PH, and a phospho-mimetic version of ROCK1 showed a loss of plasma membrane association (Ishiuchi and Takeichi, 2011). Furthermore, the p21-activated kinase 1 (Pak1), which is activated by the Par6-binding protein Cdc42, has recently been shown to act semi-redundantly with aPKC to phosphorylate shared target proteins (Figure 5A) (Aguilar-Aragon et al., 2018; Bokoch, 2003). Importantly, several putative phosphorylation sites for both Pak1 and aPKC are highly conserved between *Drosophila* and mammalian Roks (Blom et al., 1999, 2004;

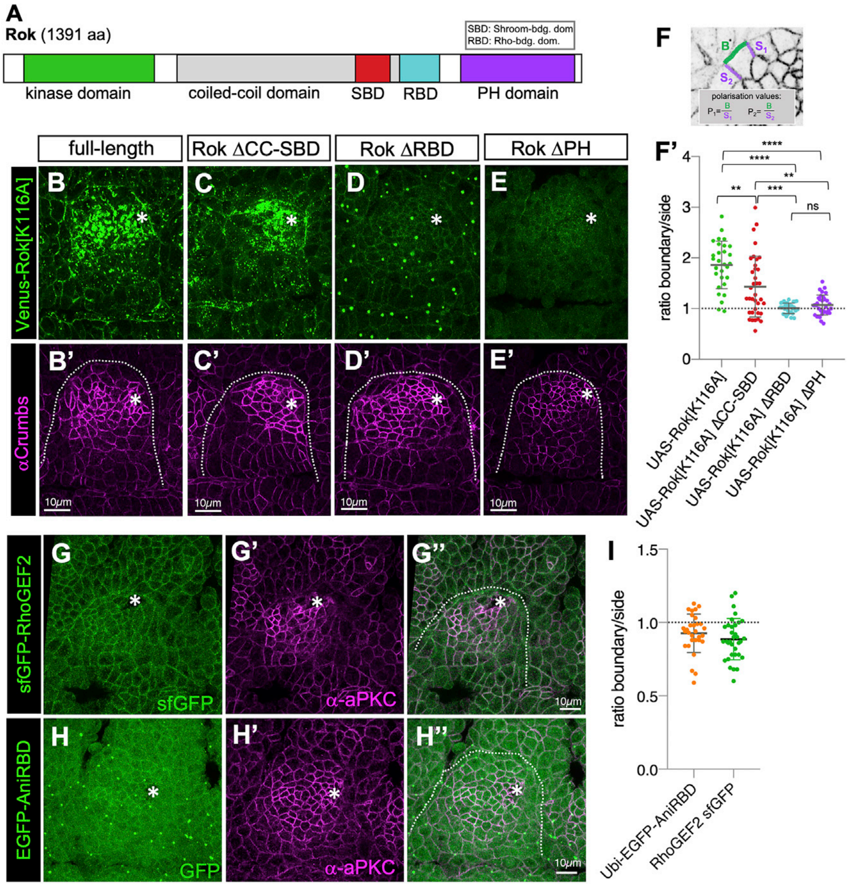

Figure 4. Rok's RBD and PH Domains Are Required for Its Planar Polarization and Placode Enrichment

(A) Schematic of Rok protein domains: N-terminal kinase domain followed by a coiled-coil domain, Shroom-binding domain (SBD), Rho-binding domain (RBD), and C-terminal PH domain.

(B–F′) Expression of variants of a Venus-tagged and kinase-dead localization reporter of Rok (*Venus-Rok[K116A]*) throughout the embryo using *Da-Gal4*: (B and B′) full-length Venus-Rok [K116A] similar to mNG-Rok shows anisotropic enrichment at the boundary (F′), though its accumulation at certain sites such as in the apical-medial region, is enhanced because of the overexpression. (C and C′) Rok lacking the coiled coil and SBD (Rok ΔCC-SBD) is less polarized at the boundary but retains some anisotropy (F′) and also shows apical-medial aggregates. (D and D′) Rok lacking the RBD (Rok ΔRBD) is still localized to cell junctions but not enriched in the placode and shows no polarization at the boundary (F′). (E and E′) Rok lacking the PH domain (Rok ΔPH) is still localized to cell junctions but not enriched in the placode and shows no polarization at the boundary (F′). (F and F′) Polarization quantification expressed as the intensity ratio of boundary junction versus side junctions (F). (F′) Mean values are as follows: rok[K116A] = 1.86, Rok ΔCC-SBD = 1.433, Rok ΔRBD = 1.005, and Rok ΔPH = 1.701. Data are represented as data points, mean, and SEM. Statistical tests used were unpaired t test; p values are indicated with ** < 0.005, *** < 0.0005, **** < 0.000; ns, not significant.

(G–I) Neither RhoGEF2 (G–G'', *sfGFP-RhoGEF2*) nor active Rho (H–H″, *EGFP-AniRBD*) are polarized at the placode boundary. (I) Polarization quantification expressed as the intensity ratio of boundary junction versus side junction. Data are represented as data points, mean, and SEM. Mean values are as follows: sfGFP-RhoGEF2 = 0.9267 and EGFP-AniRBD = 0.8862.

Rennefahrt et al., 2007) (Figures 5B and S4). We therefore set out to examine whether aPKC and/or Pak1 contributed to a regulation of Rok membrane association.

We used the GAL4/UAS system to overexpress membrane-targeted versions of aPKC or Pak1 in *enGal4* stripes in embryos with endogenously tagged mNG-Rok. While overexpression of aPKC using *UAS-aPKC[CAAX]* did not significantly affect mNG-Rok membrane localization in the embryonic epithelium compared with control cells (Figures 5C–5C″), overexpression of membrane-targeted Pak1 using *UAS-Pak1^{myr}* strongly decreased mNG-Rok membrane localization (Figures 5D–5D″).

Mass-spectrometric analysis of mammalian EpH4 tissue culture cell lysates performed by Ishiuchi and Takeichi (2011) identified nine phosphorylated sites in human ROCK1 in these cells, four of which are conserved in *Drosophila* Rok. Strikingly, all four sites are located in the Rok C-terminal region, close to the RBD and PH (Figures 4 and 5B). Furthermore, all are recognized as putative phosphorylation substrates for Pak1 and aPKC (Figures 5B and S4). We designed three short peptides covering these putative sites and performed *in vitro* kinase assays with the purified kinase domains of human Pak1 and human PKCι, using a small Bazooka peptide (BazS980) as a positive control (Figure 5E). Both Pak1 and aPKC kinase domains strongly phosphorylated the control Bazooka peptide, but no aPKC phosphorylation of any of the Rok peptides was detected. By contrast, Pak1 strongly phosphorylated Rok peptide 2 (Rok Pep2), containing serine S1365 and threonine's T1366 and T1369, which are all located close to the C-terminal end of the PH (Figures 5B and 5E). This phosphorylation was completely abolished in a peptide that had these three residues, S1365, T1366, and T1369, mutated to alanine (Figure 5E, Rok pep2AAA).

Thus, Pak1 negatively regulates Rok membrane accumulation by phosphorylating its C-terminal region.

Phosphorylation of Rok Contributes to Its Planar Polarization *In Vivo*

To confirm that phosphorylation of Rok by Pak1 played a role in its planar polarization *in vivo*, we used the previously generated mNG-Rok strain to mutate the four conserved putative phosphorylation sites described above as well as eight serines and threonines in close proximity (Figure 5B, asterisks) using CRISPR/Cas9 and homologous recombination repair (Figure 6A).

Although flies carrying this mNG-tagged phospho-site mutant Rok (mNG-Rok[Pmut]) (Figure 6B versus Figure 6C) were homozygous viable, a 2-fold increase in embryonic lethality (12.1% of fertilized embryos) was observed compared with the parental mNG-Rok strain (5.9% of fertilized embryos) (Figure 6D). There

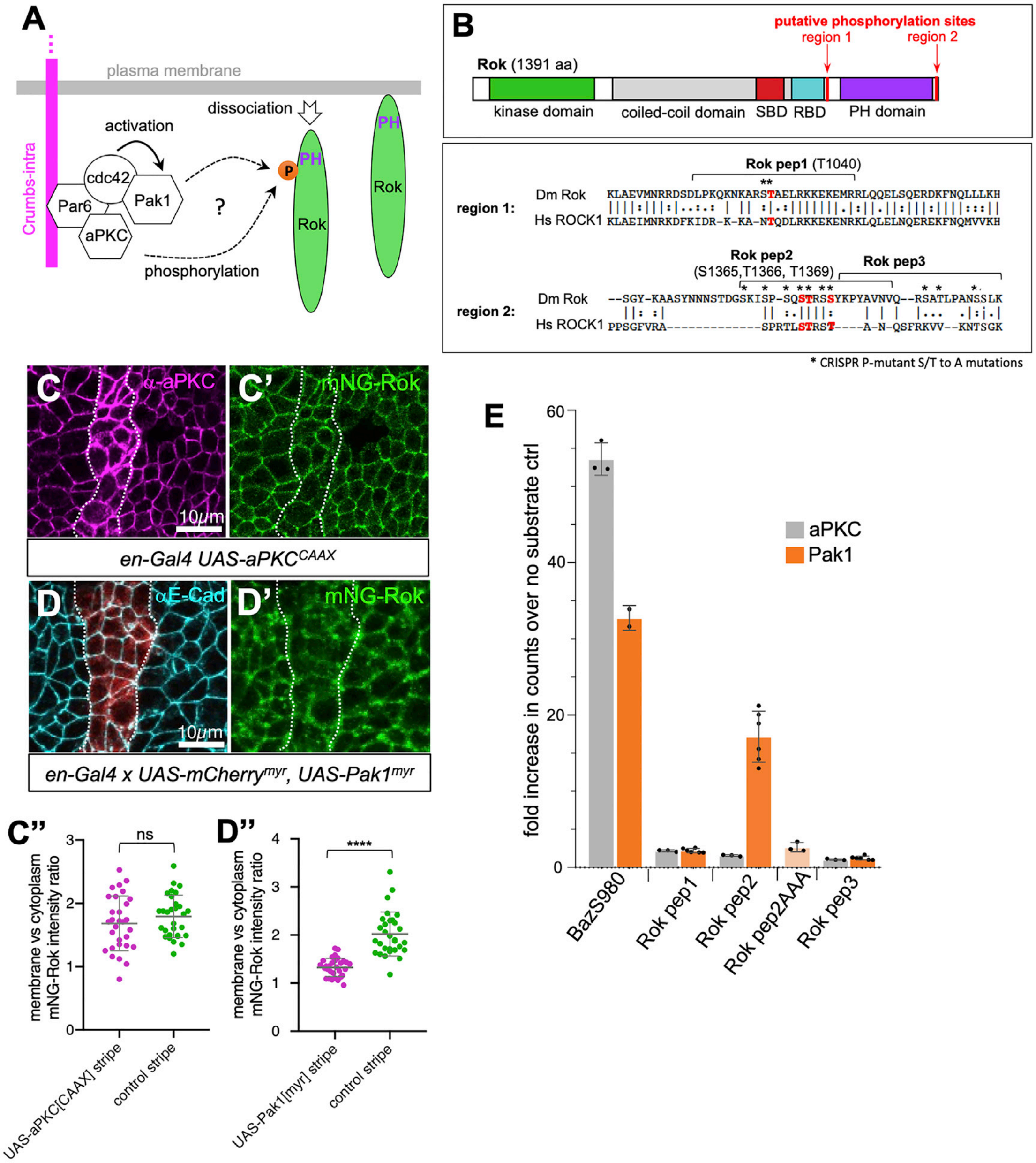

Figure 5. Pak1 Can Phosphorylate Rok and Induce Its Dissociation from the Membrane

(A) Crumbs' intracellular tail can interact with two kinases with highly overlapping phosphorylation targets: aPKC and Pak1.

(B) Both aPKC and Pak1 have many overlapping predicted phosphorylation sites in Rok, in particular near the C-terminal RBD and PH domains. Residues marked in red and named above the sequence are conserved between Dm Rok and *Hs* ROCK1 and are phosphorylated in human EpH4 cells (Ishiuchi and Takeichi, 2011). Residues marked by asterisks were mutated in mNG-Rok[Pmut], see Figure 6. Phosphorylation of Rok by either or both kinases could promote its dissociation from the plasma membrane (A). See also Figure S4.

(C–C″) Overexpression in stripes of a membrane-targeted version of aPKC (magenta in (C), *en-Gal4 UAS-aPKC[CAAX]*) does not significantly affect membrane localization of mNG-Rok (green) in the overexpressing stripes, quantified in (C″). Data are represented as data points, mean, and SEM. Unpaired t test was used to determine statistical significance; p value is 0.278 (ns).

(D–D″) Overexpression in stripes of a membrane-targeted version of Pak1 (red marks overexpression stripe in (D); *en-Gal4 x UAS-mCherry, UAS-Pak1^{myr}*) leads to a loss of mNG-Rok (green) from the membrane in the overexpressing stripes, as quantified in (D″). Data are represented as data points, mean, and SEM. Unpaired t test was used to determine statistical significance; p value is <0.0001 (****).

(E) Using purified kinase domains of either aPKC or Pak1 and short peptide substrates of Rok (indicated in B) in an *in vitro* kinase assay, we can detect phosphorylation of Rok peptide 2 (Rok pep2), which is located near the PH domain, by Pak1 but no phosphorylation of any Rok peptide tested by aPKC. Both kinase domains phosphorylate a known substrate peptide from Bazooka (BazS980). Data show fold enrichment of radioactive P(33)-phosphate counts over "no substrate" control; data points, mean, and SEM are shown. Rok pep2AAA is identical to the Rok pep2 peptide, with three potential phosphorylation sites mutated to alanine: S1365A, T1366A, and T1369A.

See also Figure S4.

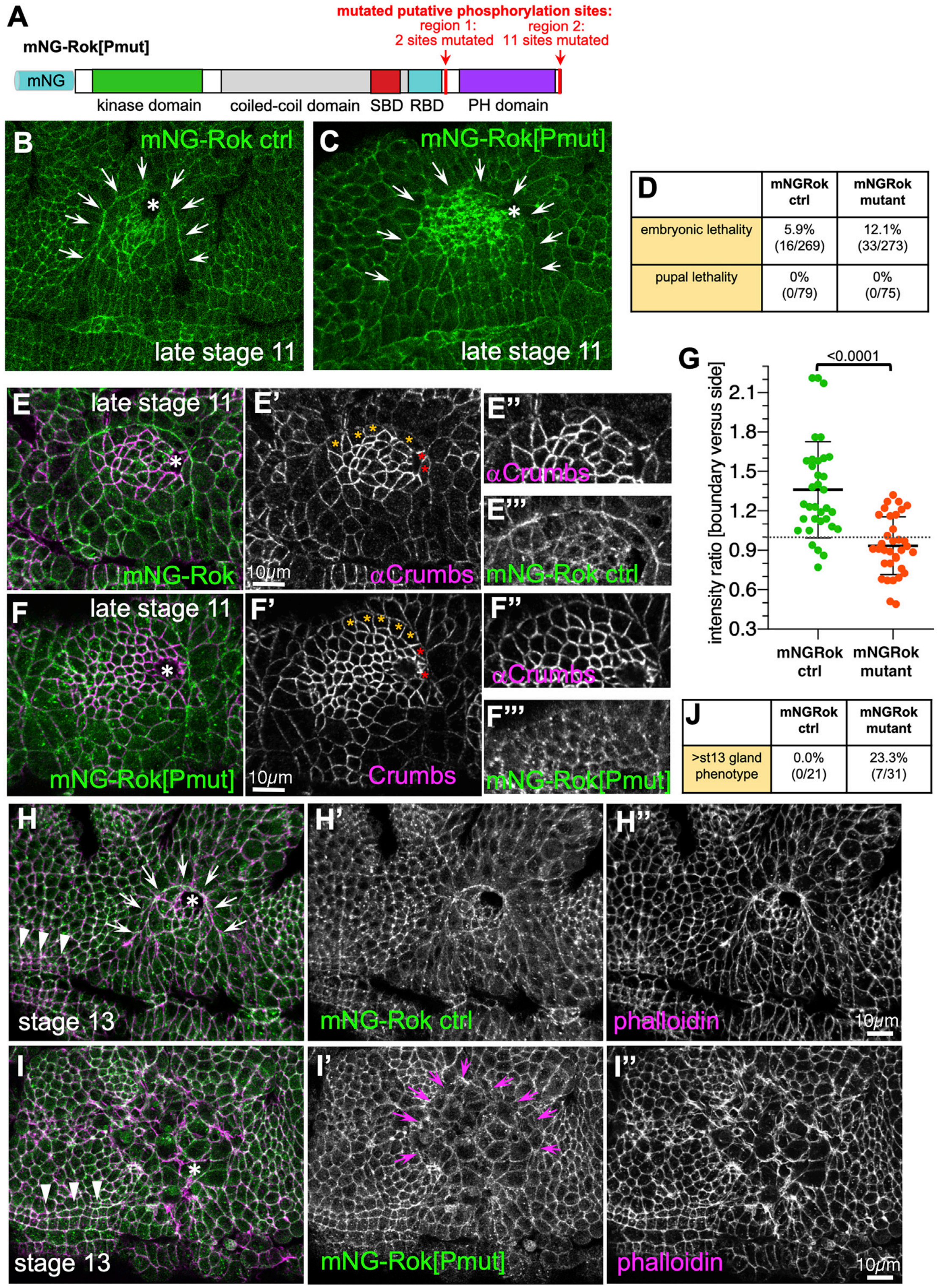

D	mNGRok ctrl	mNGRok mutant
embryonic lethality	5.9% (16/269)	12.1% (33/273)
pupal lethality	0% (0/79)	0% (0/75)

J	mNGRok ctrl	mNGRok mutant
>st13 gland phenotype	0.0% (0/21)	23.3% (7/31)

Figure 6. A Phospho-mutant Rok Shows Reduced Planar Polarization at the Boundary

(A) Schematic of the potential target sites for aPKC or Pak1 phosphorylation that are mutated in the phospho-mutant Rok (mNG-Rok[Pmut]).

(B and C) Localization comparison between mNG-Rok (B) and mNG-Rok[Pmut] (C). Arrows mark the boundary and the asterisks mark the invagination point.

(D) Flies homozygous for mNG-Rok[Pmut] are semi-viable, with 12.1% of embryos not hatching compared with 5.9% in the mNG-Rok control.

(E–G) Compared with the mNG-Rok control (E, green) that shows polarization at the boundary marked by Crumbs (magenta), mNG-Rok[Pmut] (F, green) shows a loss of polarization at the boundary marked by Crumbs (magenta). (G) Polarization quantification expressed as the intensity ratio of boundary junction versus side

(legend continued on next page)

was no increased lethality at later developmental stages. We analyzed planar polarization of mNG-Rok[Pmut] in comparison to mNG-Rok by quantifying the fluorescence intensity ratio between boundary junctions and side junctions in boundary cells that showed a clear Crumbs anisotropy (Figures 6E–6G). Although, in agreement with quantifications shown in Figure 7E, mNG-Rok showed a clear polarization at the boundary, with a mean ratio of 1.36, mNG-Rok[Pmut] was on average not polarized, with a mean ratio of 0.94 (Figure 6G). This suggests that phosphorylation of these consensus aPKC/Pak1 sites contributes to Rok planar polarization.

We could not detect any phenotype in tissue bending and salivary gland placode invagination at early stages, i.e., stages 11–12, in mNG-Rok[Pmut] embryos but could detect a fraction of embryos beyond stage 13 (23.3% in mNG-Rok[Pmut] compared with 0.0% in the mNG-Rok control) that showed a striking phenotype: we observed a disruption and altered cell shapes within the epidermis at the positions where the salivary gland placodes were located (Figures 6H–6J, arrows).The rest of the epidermis appeared largely unaffected. At this stage in control embryos, the circumferential actomyosin cable at the placode boundary was strongly enriched in F-actin (Figures 6H and 6H″, arrows) (Röper, 2012). By contrast, in these mutant mNG-Rok[P[mut] embryos, we did not detect any accumulation of F-actin at comparable junctions where the cable would be positioned in the wild type (Figures 6I and 6I″). mNG-Rok [Pmut] was still localized to junctions in the placode area, but there was no accumulation suggesting cable localization at this stage (Figure 6I′).

Thus, phosphorylation of the Rok C-terminal domain, which appears to be mediated by Pak1, is required *in vivo* for Rok planar polarization.

Pak1 Regulates Rok Planar Polarization Downstream of Crb/Cdc42

Although zygotic loss of Pak1 is lethal, maternal contribution allows embryos to develop normally as far as early stage 11, making it possible to investigate Pak1's role during salivary gland placode morphogenesis. Interestingly, Pak1 zygotic loss of function has been shown to induce defects in embryonic dorsal closure as well as in the late embryonic salivary glands (Bahri et al., 2010; Conder et al., 2007; Pirraglia et al., 2010). In early stage 11 *pak1*^{14} zygotic mutant embryos, Pak1 was still detectable in the epithelium, and early salivary placodes were not affected. However, in later placodes from late-stage 11/stage 12 onward, mNG-Rok planar polarization at the salivary gland boundary was strongly reduced (Figures 7A–7B′). Moreover, UAS-Pak1^{myr} overexpression in the salivary placode using *fkh-Gal4* (Henderson and Andrew, 2000) completely abolished mNG-Rok planar polarization at the boundary most obvious at later stages, when the placode seemed to pull away from the surrounding epithelium (Figure 7D). These results show that Pak1 plays a crucial role in salivary gland morphogenesis by regulating Rok membrane localization and planar polarization.

To understand how Crumbs might regulate Pak1 to control Rok planar polarization, we examined Pak1 protein localization in the embryonic epidermis. Similar to Crumbs protein, Pak1 was localized apically at junctions and enriched in the salivary placode (Figures 7F–7F′). Pak1 function depends on its activation by the small GTPase Cdc42 (Bokoch, 2003). An mCherry-Cdc42 reporter expressed under the control of the *sqh* promoter localized apically in junctions in the embryonic epithelium and showed anisotropic distribution at the salivary gland placode boundary similar to Crumbs (Figures 7G–7G″ and quantified in Figure 7J). Ectopic Crumbs expressed in *enGal4* stripes also recruited this Cdc42-reporter to ectopic locations (Figures 7H–7H″). In order to confirm that Cdc42 activated Pak1 in the embryonic epidermis, we examined the localization of Pak1 in cells expressing constitutively active Cdc42^{V12} (Welch et al., 1998). In stripes of cells expressing Cdc42^{V12}, Pak1 localization to junctions was strongly enhanced (Figures 7I–7I″ and 7K). Thus, the negative regulatory effect of Crumbs on Rok is likely mediated by the recruitment and activation of Pak1 by Crumbs-bound Cdc42.

We then wanted to directly test the effects of zygotic loss or overexpression of Pak1 on Rok dynamics at the membrane. To do so, we measured mNG-Rok recovery upon photobleaching (as above) in the salivary gland placode of *pak1*^{14} mutant embryos as well as in embryos overexpressing membrane-targeted Pak1 (using *UAS-Pak1*^{myr} x *fkhGal4*). Zygotic loss of Pak1 slowed the recovery of mNG-Rok in both boundary and inner junctions, whereas placodal expression of myristoylated Pak1 increased it (Figures 7L and 7M). mNG-Rok k_{off} values estimated from fluorescence recovery in *pak1*^{14} placodes (boundary junctions, 0.032 (±0.013) s^{-1} and inner junctions, 0.044 (±0.008) s^{-1}) were significantly lower than in control placodes (p value [bootstrap/boxplot], 0.0002 and 0.008, respectively), supporting our hypothesis that Pak1 promotes Rok membrane dissociation. Corroborating these data, mNG-Rok k_{off} values in placodes overexpressing Pak1^{myr} were significantly increased compared with the original control, with values of 0.170 (±0.022) s^{-1} at the boundary and 0.266 (±0.025) s^{-1} within the placode (p value [bootstrap/boxplot], 0.0002 for both; Figure 7M).

Thus, Pak1 is able to modulate Rok membrane dynamics. Altogether, our data demonstrate that Pak1 is a direct modulator of Rok dynamics responsible for Rok planar polarization downstream of Crumbs at the boundary of the salivary gland placode.

DISCUSSION

In epithelial cells, junctional proteins as well as morphogenetically active pools of actomyosin are concentrated within the apical and

junction. Data are represented as data points, mean, and SEM. Mean values are as follows: mNG-Rok ctrl, 1.36 and mNG-Rok[Pmut], 0.935. Unpaired t test was used to compare samples, and p value is indicated.

(H–J) Stage 13 mNG-Rok[Pmut] embryos show a strong disruption of the placodal boundary in 23.3% of embryos (compared with 0.0% in the control), Rok is in green in (H) and (I), with phalloidin to label cell outlines in magenta in (H) and (I).

(J) Quantification of stage 13 gland phenotype prevalence. Arrowheads in (H) and (I) point to the pharyngeal ridges and identify embryos as stage 13, arrows in (H) point to the planar polarized mNG-Rok control at the boundary, and magenta arrows in (I') point to the disorganized and disrupted boundary in mNG-Rok[Pmut].

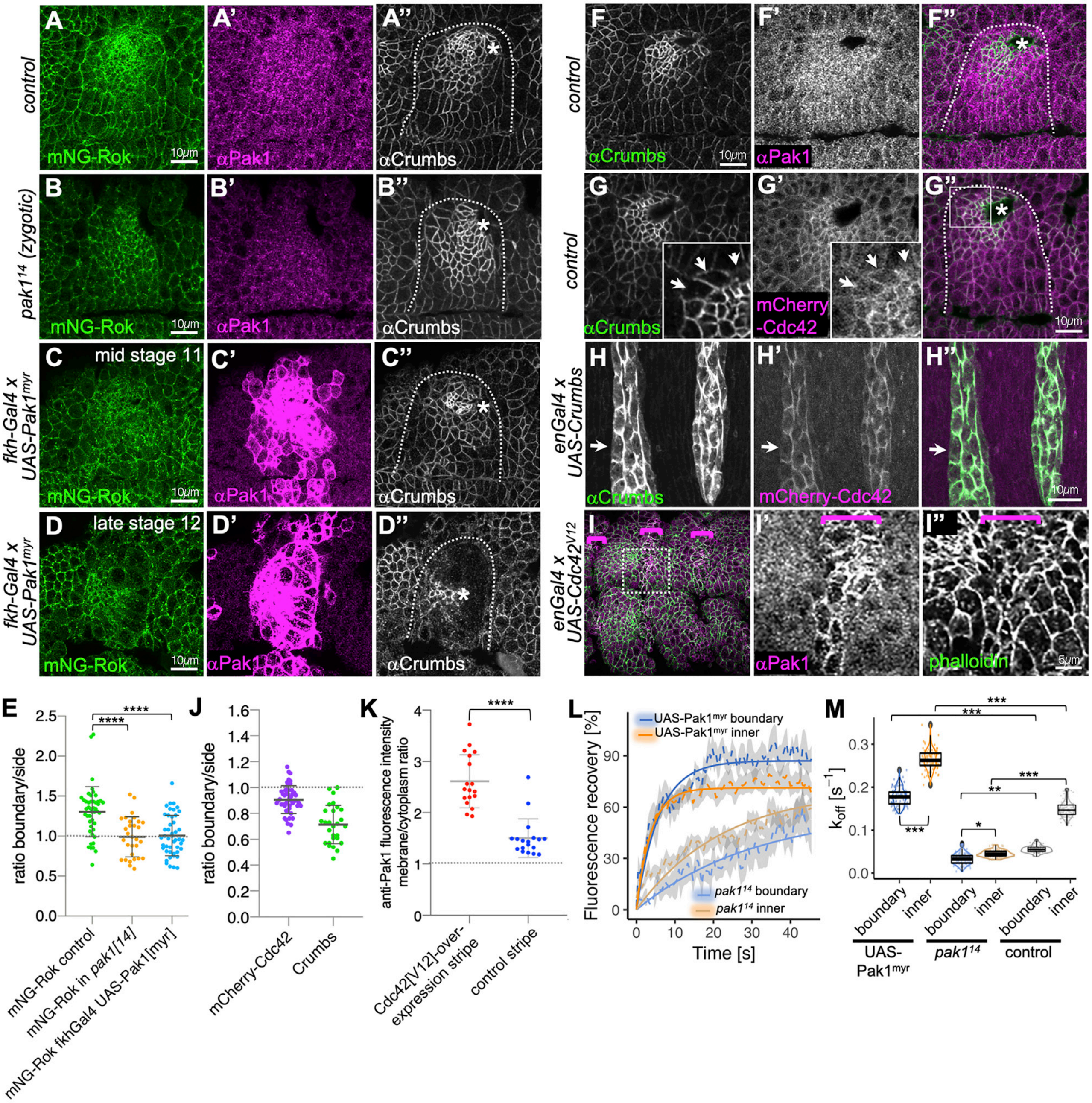

Figure 7. Pak1 Acts Downstream of Crumbs/Cdc42 and Controls Rok Planar Polarization and Cable Formation at the Placode Boundary

(A–B″) In contrast to the wild type (A–A″) where Pak1 is enriched in the placode (A′) and mNG-Rok strongly accumulates at the placode boundary (A), in *pak1*^{14} zygotic mutants Pak1 antibody labeling is strongly reduced (B′), and mNG-Rok is not polarized to the placode boundary (B, quantification in E).

(C–D″) Overexpression of the membrane-targeted form of Pak1 in the placode (*UAS-Pak1*^{myr} *x fkh-Gal4*) leads to a disorganized placode at stage 11 (C″) and a loss of mNG-Rok polarization (C, and quantification in E). At late stage 12, many *UAS-Pak1*^{myr} *x fkhGal4* placodes show a disruption that suggests a ripping of cells at the placode boundary (D″). mNG-Rok remains at remnant cell boundaries (D). (C′) and (D′) show the increased levels of overexpressed membrane-localized Pak1 by antibody staining.

(E) Polarization quantification expressed as the intensity ratio of boundary junction versus side junction. Data are represented as data points, mean, and SEM. Mean values are as follows: mNG-Rok control = 1.304, mNG-Rok in *pak1*^{14} = 0.99, and mNG-Rok in *fkh-Gal4 UAS-Pak1*myr = 1.006. Statistical tests used were unpaired t tests, and p values are indicated with **** being < 0.0001. Dotted lines indicate the placode boundary and asterisks the invagination point.

(F–F″) Pak1 (F′ and magenta, revealed by anti-Pak1 antibody) is enriched in the salivary gland placode compared with the surrounding epidermis. Crumbs is shown in (F) and green.

(G–G″) Cdc42-mCherry localization (G′ and magenta) follows endogenous Crumbs anisotropic localization (G and green) at the placode boundary; insets show magnification of boundary cells (arrows point to boundary membrane). Polarization quantification of mCherry-Cdc42 and Crumbs in corresponding cells

(legend continued on next page)

apico-lateral region of the cells. This placement is controlled by the epithelial polarity network (Tepass, 2012). Patterning of cytoskeletal activity and junctional changes, key ingredients of morphogenetic changes in epithelial tissues, then takes place within this apical domain. This can lead to apical junctional planar polarization of whole tissues or smaller domains or rows of cells that are morphogenetically active, as, for instance, is the case at the boundary of the salivary gland placode. In this case, the apical polarity determinant Crumbs plays a dual role, first in maintaining apical-basal polarity of epidermal cells including the salivary gland placode, and second in patterning cytoskeletal behavior within the apical domain. Crumbs levels, in fact, show dynamic variations across much of the *Drosophila* embryonic epidermis until stage 14, and as described here, step changes in Crumbs levels tend to be accompanied by actomyosin accumulation at these boundaries (Röper, 2013).

Rok as the key morphogenetic activator of non-muscle myosin II is crucial to development in many animals. Thus, regulation and activity of Rok in cells is closely controlled. Historic views of Rok activation assumed a potential fold-back mechanism whereby the known regulatory activity of Rok's C terminus would contact the N-terminal kinase domain and block its activity (Amano et al., 1999; Julian and Olson, 2014). An alternative view is supported by recent evidence and suggests that the C-terminal domain is crucial for membrane interaction and that Rok is in fact always found as a homodimer in an extended conformation (Truebestein et al., 2015, 2016). Such an extended conformation and role of the C terminus in membrane binding is in agreement with our *in vivo* findings that phospho-regulation of this domain is critical for membrane localization.

aPKC and Pak1 are both important kinases with a multitude of roles in development and tissue homeostasis (Hong, 2018; Rane and Minden, 2014). Their overlapping function though has only been appreciated recently (Aguilar-Aragon et al., 2018). Because of the recently published data on Pak1's and aPKC's overlapping function, we were prompted to identify Pak1 as the main effector downstream of Crumbs responsible for Rok planar polarization at the placode boundary. But why "charge" the Crumbs intracellular domain with two kinases with highly overlapping targets? The fact that Pak1 depends on Cdc42 for its activation might add another layer of control and allow differential kinase usage or amplification of kinase activity depending on the tissue context.

The differential expression level of Crumbs between salivary gland placode and surrounding epidermis, or epidermis and amnioserosa, demarcates clear boundaries. These boundaries at the plasma membrane level can then be turned into cytoskeletal planar polarization, leading to physical boundaries due to, for instance, increased tension at these boundaries. Crumbs is not the only homophilic interactor that can exert such effects. Recent examples include E- and N-Cadherin patterning during eye morphogenesis in the fly (Chan et al., 2017), the fly nectin Echinoid at clonal boundaries (Chang et al., 2011), as well as tissue-specific expression of a Cadherin, Cad2, selectively in the neural cells in *Ciona robusta*, thereby patterning myosin activity at the neural/epidermal boundary where there is a step change in Cad2 expression (Hashimoto and Munro, 2019). Interestingly, in *Ciona*, Cad2 is also titrated away from the tissue boundary because of homophilic interactions and also exerts a negative regulatory effect on myosin II accumulation. In this context though, and in contrast to the salivary gland placode boundary, it is RhoA activity that is polarized and increased at the tissue boundary because of a selective recruitment of a RhoGAP by Cad2 (Hashimoto and Munro, 2019). Thus, although common principles in patterning tissue boundaries and patterning of cytoskeletal activity are repeatedly used in development, the fine molecular details vary depending on the tissue context.

We identified a mechanism that allows for a boundary-specific regulation of Rok and myosin accumulation at membranes between differently fated groups of cells. This mechanism only occurs when a pre-existing molecular anisotropy of an upstream factor is detected, thereby circumventing an on/off negative regulatory interaction. In the *Ciona* example above, Cad2 levels are high in constricting neural cells that internalize into the embryo, very reminiscent of the situation in the salivary gland placode. Thus, the effect of Cad2 on myosin is not absolute here either. We suspect that such a mechanism of modulation of membrane residence time via affecting the k_{off} might be widely employed during tissue morphogenesis, as it allows for fine-tuning of cytoskeletal activity being integrated with other essential cell biological functions of the upstream regulators. In the case of Crumbs, its crucial role in maintaining apical-basal polarity and thereby epithelial integrity can be combined with its planar tissue patterning role.

In summary, this example of planar patterning in a morphogenetic process illustrates that the study of such processes

expressed as the intensity ratio of boundary junction versus side junction (J). Mean values are as follows: mCherry-Cdc42, 0.906 and Crumbs, 0.714. Data are represented as data points, mean, and SEM.

(H–H″) Overexpression of Crumbs (H and green) in *enGal4* stripes leads to ectopic recruitment of mCherry-Cdc42 (H′ and magenta) to sites of ectopic Crumbs, again following the anisotropy (arrows).

(I–I″) Overexpression of a constitutively active form of Cdc42, $Cdc42^{V12}$, in *enGal4* stripes leads to a strongly increased membrane association of Pak1 (magenta in I) in the overexpressing stripes. Membranes are labeled with phalloidin to reveal F-actin (green in I). (I′) and (I″) are magnifications of the box indicated in (I); data are quantified in (K).

(K) Mean values are *UAS-Cdc42*^{V12} *x enGal4* stripe, 2.61 and control stripe, 1.50. Data are represented as data points, mean, and SEM. Statistical tests used were unpaired t tests; p value is indicated with **** < 0.0001. Dotted lines indicate the boundary of the placode, and asterisks mark the invagination point.

(L and M) Modulation of Pak1 levels affects mNG-Rok k_{off}. (L) Recovery curves fitted to data of FRAP experiments for boundary and inner junctions, respectively, for Pak1 overexpression (using *UAS-Pak1*^{myr} *x fkhGal4* n[boundary], 7 and n[inner], 18) and for the *pak1*^{14} mutant (n[boundary], 5 and n[inner], 14). (M) k_{off} values were estimated from the fluorescence recovery for the boundary [*UAS-Pak1*^{myr}] as 0.17 (±0.022), for inner membranes [*UAS-Pak1*^{myr}] as 0.266 (±0.025), for the boundary [*pak1*^{14}] as 0.032 (±0.013), and for inner membranes [*pak1*^{14}] as 0.044 (±0.008). Both Pak1 modulations were found to be significantly different from the control using a bootstrap procedure, with the p values determined as *** = 0.0002, ** = 0.008, * = 0.013.

See also Figure S5.

needs to take the dynamic behavior of components into account. The current exciting advances in light microscopy, live imaging, and image quantification will be of crucial help to facilitate and transform such analyses in live tissues during development.

STAR★METHODS

Detailed methods are provided in the online version of this paper and include the following:

- KEY RESOURCES TABLE
- LEAD CONTACT AND MATERIALS AVAILABILITY
- EXPERIMENTAL MODEL
- METHOD DETAILS
    - Drosophila Stocks and Genetics
    - Generation of Transgenic Fly Lines
    - mNG-Rok Generation by CRISPR/Cas9
    - Mutagenesis of Putative *P*-sites in mNG-Rok
    - Embryo Immunofluorescence
    - Confocal, and Time-lapse imaging
    - Embryo Viability Assay
    - Rho-kinase Sequence Analysis
    - *In Vitro* Kinase Assay
- QUANTIFICATION AND STATISTICAL ANALYSIS
    - Fluorescence Intensity Quantifications
    - FRAP Imaging and Analysis
    - FRAP Curve Fitting and Statistical Analysis
    - Reasoning for k_{off} Deduction from FRAP Recovery Curves
- DATA AND CODE AVAILABILITY
    - *In Silico* Rok Particle Simulation

SUPPLEMENTAL INFORMATION

Supplemental Information can be found online at https://doi.org/10.1016/j.devcel.2019.12.003.

ACKNOWLEDGMENTS

The authors would like to thank the following people for reagents and fly stocks: Debbie Andrew, Jennifer Zallen, Tony Harris, Nicholas Harden, Barry Thompson, and Norbert Perrimon. L.J. would like to thank Simon Bullock for support. C.S. would like to thank Barry Thompson for input. The following work is supported by the Medical Research Council, UK: Röper lab (K.R. and C.S., file reference number U105178780), L.J. (file reference number MC_U105178790), J.B. (file reference number MC_U105184326), and T.J.S. (file reference number MC_U105178783).

AUTHOR CONTRIBUTIONS

Conceptualization, K.R. and C.S.; Methodology, K.R., C.S., J.B., and T.J.S.; Software, J.B. and T.J.S.; Investigation, K.R. and C.S.; Resources, C.S. and L.J.; Writing – Original Draft, K.R. and C.S.; Funding Acquisition, K.R.

DECLARATION OF INTERESTS

The authors declare no competing interests.

Received: April 20, 2019
Revised: October 24, 2019
Accepted: December 9, 2019
Published: January 2, 2020

REFERENCES

Aguilar-Aragon, M., Elbediwy, A., Foglizzo, V., Fletcher, G.C., Li, V.S.W., and Thompson, B.J. (2018). Pak1 kinase maintains apical membrane identity in epithelia. Cell Rep. *22*, 1639–1646.

Amano, M., Chihara, K., Nakamura, N., Kaneko, T., Matsuura, Y., and Kaibuchi, K. (1999). The COOH terminus of Rho-kinase negatively regulates rho-kinase activity. J. Biol. Chem. *274*, 32418–32424.

Amano, M., Nakayama, M., and Kaibuchi, K. (2010). Rho-kinase/ROCK: a key regulator of the cytoskeleton and cell polarity. Cytoskeleton (Hoboken) *67*, 545–554.

Bahri, S., Wang, S., Conder, R., Choy, J., Vlachos, S., Dong, K., Merino, C., Sigrist, S., Molnar, C., Yang, X., et al. (2010). The leading edge during dorsal closure as a model for epithelial plasticity: Pak is required for recruitment of the Scribble complex and septate junction formation. Development *137*, 2023–2032.

Bailey, M.J., and Prehoda, K.E. (2015). Establishment of Par-polarized cortical domains via phosphoregulated membrane motifs. Dev. Cell *35*, 199–210.

Bischof, J., Björklund, M., Furger, E., Schertel, C., Taipale, J., and Basler, K. (2013). A versatile platform for creating a comprehensive UAS-ORFeome library in Drosophila. Development *140*, 2434–2442.

Blom, N., Gammeltoft, S., and Brunak, S. (1999). Sequence and structure-based prediction of eukaryotic protein phosphorylation sites. J. Mol. Biol. *294*, 1351–1362.

Blom, N., Sicheritz-Pontén, T., Gupta, R., Gammeltoft, S., and Brunak, S. (2004). Prediction of post-translational glycosylation and phosphorylation of proteins from the amino acid sequence. Proteomics *4*, 1633–1649.

Bokoch, G.M. (2003). Biology of the p21-activated kinases. Annu. Rev. Biochem. *72*, 743–781.

Booth, A.J.R., Blanchard, G.B., Adams, R.J., and Röper, K. (2014). A dynamic microtubule cytoskeleton directs medial actomyosin function during tube formation. Dev. Cell *29*, 562–576.

Brand, A.H., and Perrimon, N. (1993). Targeted gene expression as a means of altering cell fates and generating dominant phenotypes. Development *118*, 401–415.

Bulgakova, N.A., and Knust, E. (2009). The Crumbs complex: from epithelial-cell polarity to retinal degeneration. J. Cell Sci. *122*, 2587–2596.

Bulinski, J.C., Odde, D.J., Howell, B.J., Salmon, T.D., and Waterman-Storer, C.M. (2001). Rapid dynamics of the microtubule binding of ensconsin in vivo. J. Cell Sci. *114*, 3885–3897.

Calzolari, S., Terriente, J., and Pujades, C. (2014). Cell segregation in the vertebrate hindbrain relies on actomyosin cables located at the interhombomeric boundaries. EMBO J. *33*, 686–701.

J. Castelli-Gair Hombria, and P. Bovolenta, eds. (2016). Organogenetic Gene Networks (Springer).

Chan, E.H., Chavadimane Shivakumar, P., Clément, R., Laugier, E., and Lenne, P. (2017). Patterned cortical tension mediated by N-cadherin controls cell geometric order in the *Drosophila* eye. eLife *6*, e22796.

Chang, L.H., Chen, P., Lien, M.T., Ho, Y.H., Lin, C.M., Pan, Y.T., Wei, S.Y., and Hsu, J.C. (2011). Differential adhesion and actomyosin cable collaborate to drive Echinoid-mediated cell sorting. Development *138*, 3803–3812.

Conder, R., Yu, H., Zahedi, B., and Harden, N. (2007). The serine/threonine kinase dPak is required for polarized assembly of F-actin bundles and apical-basal polarity in the Drosophila follicular epithelium. Dev. Biol. *305*, 470–482.

Dahmann, C., and Basler, K. (1999). Compartment boundaries: at the edge of development. Trends Genet. *15*, 320–326.

modENCODE Consortium, Roy, S., Ernst, J., Kharchenko, P.V., Kheradpour, P., Negre, N., Eaton, M.L., Landolin, J.M., Bristow, C.A., Ma, L., et al. (2010). Identification of functional elements and regulatory circuits by Drosophila modENCODE. Science *330*, 1787–1797.

Fletcher, G.C., Lucas, E.P., Brain, R., Tournier, A., and Thompson, B.J. (2012). Positive feedback and mutual antagonism combine to polarize Crumbs in the Drosophila follicle cell epithelium. Curr. Biol. *22*, 1116–1122.

Galea, G.L., Cho, Y.J., Galea, G., Molè, M.A., Rolo, A., Savery, D., Moulding, D., Culshaw, L.H., Nikolopoulou, E., Greene, N.D.E., and Copp, A.J. (2017). Biomechanical coupling facilitates spinal neural tube closure in mouse embryos. Proc. Natl. Acad. Sci. USA *114*, E5177–E5186.

Girdler, G.C., and Röper, K. (2014). Controlling cell shape changes during salivary gland tube formation in Drosophila. Semin. Cell Dev. Biol. *31*, 74–81.

Harden, N., Lee, J., Loh, H.Y., Ong, Y.M., Tan, I., Leung, T., Manser, E., and Lim, L. (1996). A Drosophila homolog of the Rac- and Cdc42-activated serine/threonine kinase PAK is a potential focal adhesion and focal complex protein that colocalizes with dynamic actin structures. Mol. Cell. Biol. *16*, 1896–1908.

Hashimoto, H., and Munro, E. (2019). Differential expression of a classic cadherin directs tissue-level contractile asymmetry during neural tube closure. Dev. Cell *51*, 158–172.e4.

Henderson, K.D., and Andrew, D.J. (2000). Regulation and function of Scr, exd, and hth in the Drosophila salivary gland. Dev. Biol. *217*, 362–374.

Hong, Y. (2018). aPKC: the kinase that phosphorylates cell polarity [version 1; peer review: 2 approved]. F1000Res. *7*, 903.

Huang, J., Zhou, W., Watson, A.M., Jan, Y.N., and Hong, Y. (2008). Efficient ends-out gene targeting in Drosophila. Genetics *180*, 703–707.

Ishiuchi, T., and Takeichi, M. (2011). Willin and Par3 cooperatively regulate epithelial apical constriction through aPKC-mediated ROCK phosphorylation. Nat. Cell Biol. *13*, 860–866.

Jacinto, A., Wood, W., Woolner, S., Hiley, C., Turner, L., Wilson, C., Martinez-Arias, A., and Martin, P. (2002). Dynamic analysis of actin cable function during Drosophila dorsal closure. Curr. Biol. *12*, 1245–1250.

Julian, L., and Olson, M.F. (2014). Rho-associated coiled-coil containing kinases (ROCK): structure, regulation, and functions. Small GTPases *5*, e29846.

Kerman, B.E., Cheshire, A.M., Myat, M.M., and Andrew, D.J. (2008). Ribbon modulates apical membrane during tube elongation through Crumbs and Moesin. Dev. Biol. *320*, 278–288.

Lowe, N., Rees, J.S., Roote, J., Ryder, E., Armean, I.M., Johnson, G., Drummond, E., Spriggs, H., Drummond, J., Magbanua, J.P., et al. (2014). Analysis of the expression patterns, subcellular localisations and interaction partners of Drosophila proteins using a pigP protein trap library. Development *141*, 3994–4005.

Major, R.J., and Irvine, K.D. (2005). Influence of Notch on dorsoventral compartmentalization and actin organization in the Drosophila wing. Development *132*, 3823–3833.

Major, R.J., and Irvine, K.D. (2006). Localization and requirement for Myosin II at the dorsal-ventral compartment boundary of the Drosophila wing. Dev. Dyn. *235*, 3051–3058.

Mason, F.M., Xie, S., Vasquez, C.G., Tworoger, M., and Martin, A.C. (2016). RhoA GTPase inhibition organizes contraction during epithelial morphogenesis. J. Cell Biol. *214*, 603–617.

Monier, B., Pélissier-Monier, A., Brand, A.H., and Sanson, B. (2010). An actomyosin-based barrier inhibits cell mixing at compartmental boundaries in Drosophila embryos. Nat. Cell Biol. *12*, 60–69.

Munjal, A., Philippe, J.M., Munro, E., and Lecuit, T. (2015). A self-organized biomechanical network drives shape changes during tissue morphogenesis. Nature *524*, 351–355.

Myat, M.M., and Andrew, D.J. (2002). Epithelial tube morphology is determined by the polarized growth and delivery of apical membrane. Cell *111*, 879–891.

Nakamura, M., Verboon, J.M., and Parkhurst, S.M. (2017). Prepatterning by RhoGEFs governs Rho GTPase spatiotemporal dynamics during wound repair. J. Cell Biol. *216*, 3959–3969.

Paré, A.C., Vichas, A., Fincher, C.T., Mirman, Z., Farrell, D.L., Mainieri, A., and Zallen, J.A. (2014). A positional Toll receptor code directs convergent extension in Drosophila. Nature *515*, 523–527.

Pirraglia, C., Walters, J., and Myat, M.M. (2010). Pak1 control of E-cadherin endocytosis regulates salivary gland lumen size and shape. Development *137*, 4177–4189.

Port, F., Chen, H.M., Lee, T., and Bullock, S.L. (2014). Optimized CRISPR/Cas tools for efficient germline and somatic genome engineering in Drosophila. Proc. Natl. Acad. Sci. USA *111*, E2967–E2976.

Ramkumar, N., Omelchenko, T., Silva-Gagliardi, N.F., McGlade, C.J., Wijnholds, J., and Anderson, K.V. (2016). Crumbs2 promotes cell ingression during the epithelial-to-mesenchymal transition at gastrulation. Nat. Cell Biol. *18*, 1281–1291.

Rane, C.K., and Minden, A. (2014). P21 activated kinases: structure, regulation, and functions. Small GTPases *5*, e28003.

Rennefahrt, U.E., Deacon, S.W., Parker, S.A., Devarajan, K., Beeser, A., Chernoff, J., Knapp, S., Turk, B.E., and Peterson, J.R. (2007). Specificity profiling of Pak kinases allows identification of novel phosphorylation sites. J. Biol. Chem. *282*, 15667–15678.

Röper, K. (2012). Anisotropy of Crumbs and aPKC drives myosin cable assembly during tube formation. Dev. Cell *23*, 939–953.

Röper, K. (2013). Supracellular actomyosin assemblies during development. Bioarchitecture *3*, 45–49.

Royou, A., Field, C., Sisson, J.C., Sullivan, W., and Karess, R. (2004). Reassessing the role and dynamics of nonmuscle myosin II during furrow formation in early Drosophila embryos. Mol. Biol. Cell *15*, 838–850.

Sanchez-Corrales, Y.E., Blanchard, G.B., and Röper, K. (2018). Radially patterned cell behaviours during tube budding from an epithelium. eLife *7*, e35717.

Sarov, M., Barz, C., Jambor, H., Hein, M.Y., Schmied, C., Suchold, D., Stender, B., Janosch, S., K J, V.V., Krishnan, R.T., et al. (2016). A genome-wide resource for the analysis of protein localisation in Drosophila. eLife *5*, e12068.

Shaner, N.C., Lambert, G.G., Chammas, A., Ni, Y., Cranfill, P.J., Baird, M.A., Sell, B.R., Allen, J.R., Day, R.N., Israelsson, M., et al. (2013). A bright monomeric green fluorescent protein derived from *Branchiostoma lanceolatum*. Nat. Methods *10*, 407–409.

Sidor, C., and Röper, K. (2016). Genetic control of salivary gland tubulogenesis in Drosophila. In Organogenetic Gene Networks, J. Castelli-Gair Hombría and P. Bovolenta, eds. (Springer International Publishing), pp. 125–149.

Simões, Sde M., Blankenship, J.T., Weitz, O., Farrell, D.L., Tamada, M., Fernandez-Gonzalez, R., and Zallen, J.A. (2010). Rho-kinase directs Bazooka/Par-3 planar polarity during Drosophila axis elongation. Dev. Cell *19*, 377–388.

Simões, Sde M., Mainieri, A., and Zallen, J.A. (2014). Rho GTPase and Shroom direct planar polarized actomyosin contractility during convergent extension. J. Cell Biol. *204*, 575–589.

Sotillos, S., Díaz-Meco, M.T., Caminero, E., Moscat, J., and Campuzano, S. (2004). DaPKC-dependent phosphorylation of Crumbs is required for epithelial cell polarity in Drosophila. J. Cell Biol. *166*, 549–557.

Sprague, B.L., Pego, R.L., Stavreva, D.A., and McNally, J.G. (2004). Analysis of binding reactions by fluorescence recovery after photobleaching. Biophys. J. *86*, 3473–3495.

Tepass, U. (2012). The apical polarity protein network in Drosophila epithelial cells: regulation of polarity, junctions, morphogenesis, cell growth, and survival. Annu. Rev. Cell Dev. Biol. *28*, 655–685.

Tepass, U., Godt, D., and Winklbauer, R. (2002). Cell sorting in animal development: signalling and adhesive mechanisms in the formation of tissue boundaries. Curr. Opin. Genet. Dev. *12*, 572–582.

Tetley, R.J., Blanchard, G.B., Fletcher, A.G., Adams, R.J., and Sanson, B. (2016). Unipolar distributions of junctional Myosin II identify cell stripe boundaries that drive cell intercalation throughout Drosophila axis extension. eLife *5*, e12094.

Truebestein, L., Elsner, D.J., Fuchs, E., and Leonard, T.A. (2015). A molecular ruler regulates cytoskeletal remodelling by the Rho kinases. Nat. Commun. *6*, 10029.

Truebestein, L., Elsner, D.J., and Leonard, T.A. (2016). Made to measure - keeping Rho kinase at a distance. Small GTPases *7*, 82–92.

Welch, H., Eguinoa, A., Stephens, L.R., and Hawkins, P.T. (1998). Protein kinase B and rac are activated in parallel within a phosphatidylinositide 3OH-kinase-controlled signaling pathway. J. Biol. Chem. *273*, 11248–11256.

Wodarz, A., Hinz, U., Engelbert, M., and Knust, E. (1995). Expression of Crumbs confers apical character on plasma membrane domains of ectodermal epithelia of Drosophila. Cell *82*, 67–76.

Zhou, B., Bagri, A., and Beckendorf, S.K. (2001). Salivary gland determination in Drosophila: a salivary-specific, fork head enhancer integrates spatial pattern and allows fork head autoregulation. Dev. Biol. *237*, 54–67.

Zou, J., Wang, X., and Wei, X. (2012). Crb apical polarity proteins maintain zebrafish retinal cone mosaics via intercellular binding of their extracellular domains. Dev. Cell *22*, 1261–1274.

STAR★METHODS

KEY RESOURCES TABLE

REAGENT or RESOURCE	SOURCE	IDENTIFIER
Antibodies		
Mouse anti-Crumbs	Developmental Studies Hybridoma Bank at the University of Iowa (DSHB)	DSHB Cat#Cq4; RRID: AB_528181
Rat anti-E-Cadherin	Developmental Studies Hybridoma Bank at the University of Iowa (DSHB)	DSHB Cat#5D3; RRID: AB_528116
anti-Pak1	(Harden et al., 1996)	N/A
Rabbit anti-aPKC (C-20)	Santa Cruz	Cat#SC-216
Rhodamine-phalloidin	Molecular Probes	Cat#R-4118
Chemicals, Peptides, and Recombinant Proteins		
EM-grade formaldehyde	Thermo Scientific	Cat#28908
γ-P33 ATP	Hartmann Analytic GmbH	N/A
P81 phosphocellulose paper	Millipore	Cat#619175
Ultima Gold XR, liquid scintillation cocktail	Perkin Elmer	Cat#6013119
BazpepS980: EHFSRDALGRR**S**ISEKHHAAL	Biomatik	N/A
Rokpep1T: LPKQKNKARS**T**AELRKKEKEM	Biomatik	N/A
Rokpep2STS: SKISPSQ**ST**RS**S**YKPYAVNV	Biomatik	N/A
Rokpep2AAA: SKISPSQ**AA**RS**A**YKPYAVNV	Biomatik	N/A
Rokpep3S: KPYAVNVQRSATLPANSSLK	Biomatik	N/A
Recombinant Pak1 kinase domain	AbNOVA	Cat#P5382
Recombinant human PKCι kinase domain	N.McDonald/B.Thompson	N/A
Experimental Models: Organisms/Strains		
Drosophila melanogaster: sqhAX3; sqh::sqhGFP42	(Royou et al., 2004)	N/A
Drosophila melanogaster: Daughterless-Gal4	Drosophila Bloomington Stock Centre	Cat#27608
Drosophila melanogaster: enGal4	Drosophila Bloomington Stock Centre	Cat#1973
Drosophila melanogaster: fkhGal4	(Henderson and Andrew, 2000; Zhou et al., 2001)	N/A
Drosophila melanogaster: Crb-GFP	(Huang et al., 2008)	N/A
Drosophila melanogaster: Zip-YFP	(Lowe et al., 2014)	N/A
Drosophila melanogaster: UAS-Crb	(Wodarz et al., 1995)	N/A
Drosophila melanogaster: *UAS-Venus-Rok*[K116A]	(Simões et al., 2014)	N/A
Drosophila melanogaster: UAS-Venus-Rok[K116A]ΔRBD	(Simões et al., 2014)	N/A
Drosophila melanogaster: UAS-Venus-Rok[K116A] ΔCC-SBD	(Simões et al., 2014)	N/A
Drosophila melanogaster: UAS-Venus-Rok[K116A]ΔPH	(Simões et al., 2014)	N/A
Drosophila melanogaster: Ubi-EGFP-AnillinRBD	(Munjal et al., 2015)	N/A
Drosophila melanogaster: sfGFP-RhoGEF2	(Sarov et al., 2016)	N/A
Drosophila melanogaster: UAS-aPKC[CAAX]	(Sotillos et al., 2004)	N/A
Drosophila melanogaster: UAS-Pak1[myr]	Drosophila Bloomington Stock Centre	Cat#8804
Drosophila melanogaster: UAS-Cdc42[V12]	Drosophila Bloomington Stock Centre	Cat#4854
Drosophila melanogaster: sqh::Cdc42-mCherry	Drosophila Bloomington Stock Centre	Cat#42236
Drosophila melanogaster: pak1[14]	Drosophila Bloomington Stock Centre	Cat#9123
Drosophila melanogaster: y[1] sc[1] v[1]; [y[+t7.7] v[+t1.8]=nanos-Cas9]attp2	Drosophila Bloomington Stock Centre	Cat#78782
Drosophila melanogaster: mNG-Rok	This paper	N/A
Drosophila melanogaster: mNG-Rok[phospho mut]	This paper	N/A

(*Continued on next page*)

Continued		
REAGENT or RESOURCE	SOURCE	IDENTIFIER
Oligonucleotides		
gRNA 1: GACCAACAGGAAGCAGCAGCTGG	Sigma Aldrich	N/A
gRNA2: GCGCCGGTGAGTGCACGAGATGG	Sigma Aldrich	N/A
P56F: 5''-GTCGACCAACAGGAAGCAGCAGC-3'	Sigma Aldrich	N/A
P56R: 5'-AAACGCTGCTGCTTCCTGTTGGT-3'	Sigma Aldrich	N/A
P57F: 5'-GTCGCGCCGGTGAGTGCACGAGA-3'	Sigma Aldrich	N/A
P57R: 5'-AAACTCTCGTGCACTCACCGGCG-3'	Sigma Aldrich	N/A
P51F: 5'GACGGTATCGATAAGCTTGATATCGG CGCAGCGTCTAATTGAAAC-3'	Sigma Aldrich	N/A
P51R: 5'GCTGATACTGCTGCT**aCA**GCTGCTGC-3'	Sigma Aldrich	N/A
5'TGCCAGCTGGACGAGAAACTGTGACCAAG CAGCGCAGCATGGATGTGGAACGAAGGCGC CGgtgagtgcacgaga**tgt**cggcccaaaagc	Sigma Aldrich	N/A
P54F: 5'GGAAGCAGCAGC**TGt**AGCAGCAGTA TCAGCTTGTTATCTTGCATTTGCATGGTGAGC AAGGGCGAGGAG-3'	Sigma Aldrich	N/A
P54R: 5'GCTTGGTCACAGTTTCTCGTCCAGCT GGCATGCCGGATCCGCCGCCCGATCCGC CGCCGGATCCGCCCTTGTAAAGTTCATCCAT CCCC-3'	Sigma Aldrich	N/A
Recombinant DNA		
pCFD3 vector	Addgene	Cat#49410
mNeonGreen vector	Allele Biotechnology & Pharmaceuticals/ (Shaner et al., 2013)	N/A
Software and Algorithms		
ImageJ/Fiji	NIH	N/A
Imaris	Bitplane	N/A
Python code for 2D particle simulation	This paper/ https://github.com/tjs23/memodis	N/A

LEAD CONTACT AND MATERIALS AVAILABILITY

Further information and requests for resources and reagents should be directed to and will be fulfilled by the lead contact, Dr. Katja Röper (kroeper@mrc-lmb.cam.ac.uk).

EXPERIMENTAL MODEL

Drosophila melanogaster was cultured using standard techniques at 25°C (unless otherwise noted); both male and female animals were used.

METHOD DETAILS

Drosophila Stocks and Genetics

The following transgenic fly lines were used and are also listed in the Key Resources Table: *sqhAX3; sqh::sqhGFP42* (Royou et al., 2004), *Daughterless-Gal4* and *enGal4* (Bloomington Stock Centre); *fkhGal4* [(Henderson and Andrew, 2000; Zhou et al., 2001) kind gift of Debbie Andrew]; *Crb-GFP* (Huang et al., 2008); *Zip-YFP* (Lowe et al., 2014); *UAS-Crb* (Wodarz et al., 1995); UAS-Venus-Rok [K116A], UAS-Venus-Rok[K116A]ΔRBD, UAS-Venus-Rok[K116A] ΔCC-SBD; UAS-Venus-Rok[K116A]ΔPH (Simões et al., 2014); *Ubi-EGFP-AnillinRBD* (Munjal et al., 2015); *sfGFP-RhoGEF2* (Sarov et al., 2016); *UAS-aPKC[CAAX]* (Sotillos et al., 2004); *UAS-Pak1^{myr}*; *UAS-Cdc42[V12]*; *sqh::Cdc42-mCherry* (Bloomington Stock Centre); *pak1[14]* (gift from B.Thompson); *y[1] sc[1] v[1]; [y[+t7.7] v[+t1.8] = nanos-Cas9]attp2* (gift from N. Perrimon).

Genotypes analysed are indicated in the figure panels and legends.

Generation of Transgenic Fly Lines

To generate *Drosophila rok* transgenic lines, donor (150 ng/μl) and guide RNA (100 ng/μl) plasmids were injected in pools (Bischof et al., 2013) into *nanos::Cas9* (chromosome 3) embryos for the endogenous tagging, or into *mNG-Rok* ; *nanos::Cas9* embryos for the generation of the phospho-site mutant.

mNG-Rok Generation by CRISPR/Cas9

Two gRNAs targeting loci near the start codon of the Rho kinase gene were cloned into pCFD3 vector (Addgene 49410) following the protocol from (Port et al., 2014). A step by step protocol is available at (www.crisprflydesign.org).

Sequences of the guide RNAs were as follows:
gRNA 1: GACCAACAGGAAGCAGCAGCTGG
gRNA2: GCGCCGGTGAGTGCACGAGATGG
PCR primers for cloning into pCFD3:
P56F: 5'-GTCGACCAACAGGAAGCAGCAGC-3''
P56R: 5'-AAACGCTGCTGCTTCCTGTTGGT-3'
P57F: 5'-GTCGCGCCGGTGAGTGCACGAGA-3'
P57R: 5'-AAACTCTCGTGCACTCACCGGCG-3'

A donor construct containing the mNeonGreen sequence in fusion with the *rok* gene in its genomic region was cloned into pBluescript SK(+) using Gibson assembly. *mNeonGreen* was cloned between two 1kb-long homology sequences corresponding to the genomic sequence on either side of the insertion site, to create homology arms for directed repair. The 1 kb regions were amplified by PCR on each side of the desired insertion site from genomic DNA. The mNeonGreen gene (Shaner et al., 2013), was amplified by PCR from the mNeonGreen vector (Allele Biotechnology), with the exclusion of the stop codon and the addition of a C-terminal linker.

Primers were designed with additional 5' sequences (underlined below) to allow triple ligation of the three PCR products into a pBluescript SK(+) vector using the Gibson Assembly Master Mix (NEB). PAM sites were mutagenized (indicated in bold below) to prevent re-cutting by Cas9 after transgenesis.

Primer sequences:
Left homology arm PCR primers:
P51F: 5'<u>GACGGTATCGATAAGCTTGATATCG</u>GCGCAGCGTCTAATTGAAAC-3'
P51R: 5'GCTGATACTGCTGCT**aCA**GCTGCTGC-3'
Right homology arm PCR primers: 5'TGCCAGCTGGACGAGAAACTGTGACCAAGCAGCGCAGCATGGATGTGGAACGAAGGCGCCGgtgagtgcacgagatgtcggcccaaaagc
mNeonGreen PCR primers:
P54F: 5'<u>GGAAGCAGCAGC</u>**TGt**<u>AGC</u>AGCAGTATCAGCTTGTTATCTTGCATTTGCATGGTGAGCAAGGGCGAGGAG-3'
P54R: 5'<u>GCTTGGTCACAGTTTCTCGTCCAGCTGGCA</u>TGCCGGATCCGCCGCCCGATCCGCCGCCGGATCCGCCCTTGTAAAGTTCATCCATCCCC-3'

Modifications were verified by sequencing of genomic DNA.

Mutagenesis of Putative *P*-sites in mNG-Rok

Guide RNAs

Four gRNAs targeting loci on both sides of the C-terminal region of Rok containing the putative phosphorylation sites to be mutated were cloned into pCFD3 vector (Addgene 49410) following the protocol from (Port et al., 2014).

Sequences: LJ20, LJ21, LJ22, LJ23

Donor Plasmid

A donor construct containing, between two 1kb-long homology arms, a Rok C-term region mutagenized on 13 selected putative phosphorylation sites, was cloned into pBluescript SK(+). The Rok C-term region was amplified by PCR and cloned into pBluescript SK(+)with primers designed to mutagenise the selected 13 putative phosphorylation sites (Figure 5B) and the 4 PAM sites.

Modifications were verified by sequencing of genomic DNA.

Embryo Immunofluorescence

Embryos were collected on apple juice-agar plates and processed for immunofluorescence using standard procedures. Briefly, embryos were dechorionated in 50% bleach, fixed in 10% EM-grade formaldehyde, and stained with primary and secondary antibodies in PBT (PBS plus 0.5% bovine serum albumin and 0.3% Triton X-100). anti-Crumbs and anti-E-Cadherin antibodies were obtained from the Developmental Studies Hybridoma Bank at the University of Iowa (DSHB); anti-Pak1 (Harden et al., 1996); anti-aPKC (Santa Cruz); rhodamine-coupled phalloidin (Molecular Probes). Secondary antibodies used were Alexa Fluor 488/Fluor 549/Fluor 649 coupled (Molecular Probes) and Cy3 and Cy5 coupled (Jackson Immuno Research Laboratories). Samples were embedded in Vectashield (Vectorlabs).

Confocal, and Time-lapse imaging

Images of fixed samples were acquired on an Olympus FluoView 1200 or a Zeiss 780 Confocal Laser scanning system as z-stacks to cover the whole apical surface of cells in the placode. Z-stack projections were assembled in ImageJ or Imaris (Bitplane), 3D rendering was performed in Imaris.

For live time-lapse experiments embryos of the genotype *Crumbs-GFP Zipper-YFP* or *mNG-Rok* were dechorionated in 50% bleach and extensively rinsed in water. Embryos were manually aligned and attached to heptane-glue coated coverslips and mounted on custom-made metal slides; embryos were covered using halocarbon oil 27 (Sigma) and viability after imaging after 24h was controlled prior to further data analysis. Time-lapse sequences were imaged under a 40x/1.3NA oil objective on an inverted Zeiss 780 Laser scanning system. Z-stack projections to generate movies in Supplemental Information were assembled in ImageJ.

Embryo Viability Assay

Embryos of the genotype *mNG-Rok* control or *mNG-Rok[Pmut]* were treated as for live imaging and mounted in separate sets of 100 embryos per experiment and let to develop at 18°C. After 48 hours hatched larvae, unfertilised embryos and developed but dead embryos were counted.

Rho-kinase Sequence Analysis

We used published predictive algorithms to identify potential aPKC and Pak1 phosphorylation sites in Dm Rok, DISPHOS (http://www.dabi.temple.edu/disphos/) and NetPhos3.1 (http://www.cbs.dtu.dk/services/NetPhos/). Rok sequences form different species were compared and aligned in EMBOSS Matcher (https://www.ebi.ac.uk/Tools/psa/emboss_matcher/).

In Vitro Kinase Assay

The following High-pressure liquid chromatography (HPLC)-purified peptides were ordered from Biomatik:

BazpepS980: EHFSRDALGRRSISEKHHAAL
Rokpep1T: LPKQKNKARSTAELRKKEKEM
Rokpep2STS: SKISPSQSTRSSYKPYAVNV
Rokpep2AAA: SKISPSQAARSAYKPYAVNV
Rokpep3S: KPYAVNVQRSATLPANSSLK

For *in vitro* kinase assays, 10μg of peptide substrate were incubated with either 150pg recombinant human Pak1 kinase domain (AbNOVA) or 0.1 μM recombinant human PKCι kinase domain (a gift from N. McDonald via B. Thompson) for 30 min at 30°C in kinase reaction buffer (50 mM HEPES [(4-(2- hydroxyethyl)-1-piperazineethanesulfonic acid] pH 7.5, 10 mM MgCl2, 1 mM EGTA, 0.01% Brij35) containing 10 μM cold ATP and 3 μCi γ-P33 ATP (Hartmann Analytic GmbH). Samples were blotted on 2cm x 2cm squares of P81 phosphocellulose paper (Millipore) and washed 3 x 10 min in 1% phosphoric acid, then 5 min in acetone. Dried papers were then transferred to scintillation vials and immersed in liquid scintillation cocktail (Ultima Gold XR, Perkin Elmer). Incorporation of γ-P33 was quantified in counts per minute by scintillation counting (Beckman LS 6500).

QUANTIFICATION AND STATISTICAL ANALYSIS

Fluorescence Intensity Quantifications

Fluorescence intensity was determined in ImageJ using projections covering the apical junctional region (as determined by Crumbs or E-Cadherin staining). Using Crumbs labelling of placodes, boundary cells showing clear Crumbs anisotropy were identified and quantified. 3- to 5-pixel-wide lines to cover the width of junctions (depending on the resolution of the image) were drawn at the boundary and at the sides of the boundary cells (see Figure 4F). The intensity was divided by the area covered for each junction to determine the intensity/pixel. Values for the boundary junction were divided by the values of the side junctions to determine two polarisation values per boundary cell.

Membrane versus cytoplasm enrichment was determined in ImageJ using projections covering the apical junctional region (as determined by Crumbs or E-Cadherin staining). 3- to 5-pixel-wide lines to cover the width of junctions (depending on the resolution of the image) were drawn at a cell junction and a comparable line was drawn across the apical cytoplasm. The intensity of each line was divided by the area covered to determine the intensity/pixel. Values for the cell junction were divided by the values of the cytoplasm to determine the membrane versus cytoplasm enrichment.

N values for quantifications are as follows: Figure 4F': *UAS-Rok[K116A]*: 4 placodes (3 embryos), 15 cells (29 polarisation values); *UAS-Rok[K116A]ΔCC-SBD*: 6 placodes (3 embryos), 18 cells (36 pol. values); *UAS-Rok[K116A]ΔRBD*: 5 placodes (3 embryos), 16 cells (32 pol. values); *UAS-Rok[K116A]ΔPH*: 6 placodes (3 embryos), 16 cells (32 pol. values)/ Figure 4I: *Ubi-EGFP-AniRBD*: 4 placodes (3 embryos), 15 cells (30 pol. values); *RhoGEF2 sfGFP*: 3 placodes (3 embryos), 17 cells (34 pol. values)/ Figure 5C'':*UAS-aPKC [CAAX]*: 3 embryos (6 overexpression stripes), 30 cells; *Control for aPKC stripe*:3 embryos (6 control stripes), 30 cells/ Figure 5D'': *UAS-Pak1[myr]*: 4 embryos (7 overexpression stripes), 35 cells; *Control for Pak1 stripe*: 4 embryos (7 control stripes), 35 cells/ Figure 6E: *mNG-Rok ctrl*: 4 placodes (2 embryos), 23 cells (46 pol. values); *mNG-Rok in pak1*[14]: 3 placodes (2 embryos), 15 cells (30 pol. values); *mNG-Rok fkhGal4 UAS-Pak1[myr]*: 4 placodes (2 embryos), 26 cells (32 pol. values)/ Figure 6J: *mCherry-Cdc42*: 4 placodes (3 embryos), 24 cells (48 pol. values); *Crumbs*: 3 placodes (2 embryos), 14 cells (28 pol. values)/ Figure 7G: *mNG-Rok ctrl*: 4 placodes (4 embryos), 17 cells (34 pol. values); *mNG-Rok[Pmut]*: 4 placodes (4 embryos), 18 cells (34 pol. measurements).

Statistical significance in comparisons was determined using unpaired t-test. Plots show data points, mean and SEM. p-values are indicated as ** being <0.005, *** being <0.0005, ****<0.0001, ns being not significant.

FRAP Imaging and Analysis

Focussing on the apical region of the salivary placode epithelium in stage 11/12 mNG-Rok embryos (or mNG-Rok in the indicated genetic backgrounds), spinning disk confocal/Fluorescence Recovery After Fluorescence (FRAP) was performed on a custom-built set-up based on a Nikon Ti stand equipped with perfect focus system, a fast Z piezo stage (ASI), a PLAN NA 1.4 60× objective and a spinning disk head (Yokogawa CSUX1) followed by 1.2x relay optics. Images were recorded with a Photometrics Prime 95B back-illuminated sCMOS camera run in pseudo global shutter mode and synchronized with the spinning disk wheel. FRAP was performed using a iLAS2 galvanometer module (Roper France) mounted on the back port of the stand and combined with the side spinning disk illumination path using a broadband polarizing beamsplitter mounted in a custom 3D-printed fluorescence filter cube. GFP was excited/bleached by a 150mW 488nm laser (Coherent OBIS mounted in a Cairn laser launch) and GFP fluorescence was imaged using a Chroma 525/50 bandpass filter. System was operated by Metamorph. About 2 μm thick z-sections (either 3 x 0.65μm or 5 x 0.5μm) were acquired to compensate for movement in z during acquisition. For the FRAP, bleach dwell time was 19ms with 28% 488nm laser power. Images were acquired at ~500ms (for 3 z steps) or ~750ms (for 5 z steps) intervals, 6 time points pre-bleach and 60 (or 45) time points post bleach.

We analysed kymographs of recovery regions and surrounding membrane regions to exclude that recovery was due to lateral movement of mNG-Rok in the membrane rather than recovery from the cytoplasmic pool.

Movies were analysed in ImageJ/Fiji (NIH). Fluorescence intensity was measured in a 6-pixel circular ROI at the site of bleach.

Measures were then normalised to account for the general photobleaching caused by image acquisition. All values were multiplied by a photobleaching correction factor determined from a 100μm diameter circle surrounding the bleach site.

$C_{photobleaching}$:

$$C_{photobleaching}(t) = \frac{background\ circle\ intensity\ at\ t1}{background\ circle\ intensity\ (t)}.$$

Normalised fluorescence intensity measurements were used to plot the percentage of fluorescence recovery after photobleaching as follows:with $F_{prebleach} = avg\ F_{t1\ to\ t6}$.

$$F(t) = \frac{(F_t - F_{postbleach})}{(F_{prebleach} - F_{postbleach})}.$$

FRAP Curve Fitting and Statistical Analysis

The k_{off} was estimated from the whole set of normalized fluorescence recovery curves. We modeled the recovery using a single exponential function in the form of: $a\ (1 - e^{-k_{off}t})$. A detailed description of the deduction of the k_{off} can be found below. A non-linear regression algorithm was used to estimate parameters from the data without prior averaging. As individual fluorescence recovery curves were noisy, we used a bootstrap procedure generating 200 estimates to estimate the statistical confidence of the estimated k_{off}. This allowed us to compute a p-value using a t-test based on bootstrap variance. Note that this p-value does not depend on the number of bootstrap samples.

Reasoning for k_{off} Deduction from FRAP Recovery Curves

Rok association and dissociation from the membrane is a binding reaction. Therefore, we decided to base our analysis on work from the McNally laboratory (Sprague et al., 2004), which presents a comprehensive and systematic approach to analysing binding characteristics from FRAP curves.

We used the following binding reaction to describe our system:

$$F + S \underset{k_{off}}{\overset{k_{on}}{\rightleftharpoons}} C.$$

This model can be formalized as a set of three reaction-diffusion equations for each quantity F (free proteins), S (vacant binding sites) and C (bound complexes) (Sprague et al., 2004):

$$\frac{d[F]}{dt} = \overbrace{D_F \nabla^2 [F]}^{Diffusion} - k_{on}[F][S] + k_{off}[C].$$

$$\frac{d[S]}{dt} = D_S \nabla^2 [S] - k_{on}[F][S] + k_{off}[C].$$

$$\frac{d[C]}{dt} = D_C \nabla^2 [C] + k_{on}[F][S] - k_{off}[C].$$

We assume the system is at equilibrium before the bleaching event, so that for t < 0

$$[S] = S_{eq},\quad [F] = F_{eq}\ \text{ and }\ [C] = C_{eq}.$$

Let us consider now the evolution of species concentrations after photo-bleaching for t > 0. First, as photo-bleaching does not affect the number of binding sites, we can assume that their concentration [S] is constant and still equal to S_{eq}. Secondly, we found that the cytoplasmic pool of mNG-Rok bleached next to the bleached membrane recovers almost immediately (data not shown). Thus, we can assume that the diffusion coefficient of free proteins F is very high and that the pool of bleached free proteins is immediately replaced so that their concentration remains constant over time and equal to F_{eq}. In addition, we found no evidence of lateral diffusion of mNG-Rok in the membrane in the time course of the experiment (data not shown). Therefore, we assume the diffusion of bound proteins C to be negligible in this context. As a consequence, the first two equations describing the evolution of the free proteins F and binding sites S are simplified and we can keep only the third equation which describes the concentration of fluorescently labelled bound proteins:

$$\frac{d[C]}{dt} = k_{on}S_{eq}F_{eq} - k_{off}[C].$$

At equilibrium, this one becomes:

$$\frac{d[C]}{dt} = 0 = k_{on}S_{eq}F_{eq} - k_{off}C_{eq}.$$

Such that we can express the on-rate-constant as:

$$k_{on} = \frac{k_{\mathrm{off}}C_{\mathrm{eq}}}{S_{\mathrm{eq}}F_{\mathrm{eq}}}.$$

We can now combine this expression with the previous equation and arrive at:

$$\frac{d[C]}{dt} = k_{\mathrm{off}}C_{eq} - k_{\mathrm{off}}[C].$$

As described previously by (Bulinski et al., 2001) and (Sprague et al., 2004) we deduce analytically the evolution of the concentration of fluorescently labelled bound proteins [C] as:

$$[C] = C_{eq}\left(1 - e^{-k_{\mathrm{off}}t}\right).$$

DATA AND CODE AVAILABILITY

In Silico Rok Particle Simulation

As illustrated in Figure 3F, we simulated the diffusion of membrane bound and unbound particles (corresponding to Rok) within a pixel representation of a placodal cell layer containing inner and boundary cells. Our intention was to create a deliberately simple two-dimensional model that might nonetheless recapitulate the salient features of the observed planar polarity. Different membrane association and dissociation constants were modelled in different regions of the simulation according to the zones illustrated in Figure 3G, i.e. distinguishing the placode boundary from the inner placode membranes including the side membranes. Here the representation of the cell membranes with superimposed particle positions automatically provided a visualization of the simulation progress. By counting bound and unbound particles in different regions, polarisation at the boundary was quantified and compared with microscopic measurements. An exhaustive grid-search for parameters, which was initially coarse and then more fine-grained, was performed to determine the combinations of diffusion, binding, unbinding and simulation time-step values that matched observations.

In detail, point particles were modelled as moving with random sequential displacements according to 2D Gaussian diffusion within a grid-based (i.e. pixel) representation of a cell layer with membrane boundaries. Particles were set to freely diffuse (off-grid) in the cell interior or diffuse laterally along the interior edge of the cell membrane; i.e. as unbound and bound sates. The positional variance for free diffusion (sigma) was set to correspond approximately to a diffusion coefficient of 25 $\mu m^2/s$ within a 25 μm wide cell area, which is the diffusion coefficient measured *in vivo* for mammalian GFP-ROCK2 (Truebestein et al., 2015). Diffusion of bound particles within the membrane was restricted to adjacent sites and was much slower than for free particles, with diffusion coefficients tested in the range 0.1 - 0.001 $\mu m^2/s$ (which made no practical difference) and final simulations set at 0.01 $\mu m^2/s$. Using a probabilistic model, that can be related to k_{on} (association) and k_{off} (dissociation) constants, free particles that collided with the cell membrane were able to bind and bound particles were able to spontaneously unbind/dissociate. Here simulation steps typically corresponded to time segments of 50 ms, though a range of values was tested. k_{off} (in units of per second) was used to set the long-term probability of each bound particle spontaneously unbinding within the simulation time-step. k_{on} was less straightforward to model as, for particles which collide with the membrane, it depends on the concentration of receptive membrane sites and this varies throughout the simulation. Accordingly, at-membrane binding probabilities of initially free particles were set dynamically so that the derived, average k_{on} measured in the simulation converged to a desired value. In essence membrane binding probability was increased when the target k_{on} was undershot and reduced when overshot, averaging around a fixed k_{on}. A single k_{on} value was used for all membrane regions, but different k_{off} values were used for placode boundary membranes and inner/side placode membranes.

The *in vivo* k_{on} used in simulations shown in Figure 3 was estimated using the following equation from the ''diffusion plus binding'' model from (Sprague et al., 2004):

$$k_{on}{}^{*} = \frac{k_{off} C_{eq}}{F_{eq}}$$

The k_{off} was calculated from our FRAP experiments, and we found that the $\frac{C_{eq}}{F_{eq}}$ ratio could be deduced from parameters measured *in vivo*:

$$\frac{C_{eq}}{F_{eq}} = \frac{[membrane]}{[cytoplasmic]} \times mobile\ fraction.$$

We measured the membrane to cytoplasmic ratio of mNG-Rok in stage 11 embryos and the value of the k_{off} and mobile fraction were calculated from fitted FRAP curves (Figures 3C, S3A, and S3B): $k_{off} = 0.149$; mobile fraction = 0.77; membrane versus cytoplasm ration: 2.367.

Using the above equation and *in vivo* measured values, we estimated the k_{on} to be about 0.27, using 0.3 for simulations in Figure 3, but also testing a range of k_{on} values between 0.1 and 0.4 in the simulations (0.2, 0.3. and 0.4 are shown). Varying k_{on} within this range had only minor effects on polarisation values.

In order that the membrane binding sites be capable of saturation, the cell edge pixels were subjected to a maximum occupancy value. For the final simulations this was set at a value of 1 particle per pixel. Occupancy limits of 1-3 particles per pixel were tested and overall this made little difference to the cell polarity, but had a notable effect on the bound/free ratio, as we might expect.

Although the number of particles modelled within each cell could be varied, this made little difference to the long term bound/unbound ratios, but naturally more particles gave smoother, less variant values. Typically, 2000 particles per cell were simulated. The particle simulation was started with randomly distributed particle positions within the cell interiors and progressed through 2000 unmonitored steps to equilibrate the model. Thereafter analyses of the particle positions were made at regularly spaced intervals for a further 10,000 steps. At each sample point the counts of bound and unbound particles were recorded for the regions marked in Figure 3G and later averaged for the whole simulation. Particle number had a slight effect on polarisation values, with smaller particle numbers leading to slightly higher polarisation values (polarisation of 1.90 for 125 particles versus polarisation of 1.48 for 2000 particles, with k[on] of 0.3 and the measured values of k[off] for both situations).

Python code to perform the 2D cell particle simulations, generating both regional counts and pixmap images, is available at: https://github.com/tjs23/memodis.

Developmental Cell, Volume *52*

Supplemental Information

Rho-Kinase Planar Polarization at Tissue Boundaries Depends on Phospho-regulation of Membrane Residence Time

Clara Sidor, Tim J. Stevens, Li Jin, Jérôme Boulanger, and Katja Röper

Figure S1, related to Figure 1. Wide-spread apical planar polarisation of Crumbs and myosin II during morphogenesis.

A-A'' Still images of a time lapse movie of embryos expressing tagged myosin II (*Zipper-YFP*, green) and Crumbs (*Crumbs-GFP*, magenta), Fig.1A' shows t=0 min and t=99 min of this movie, intervening time points are displayed here to illustrate the morphogenetic process.

B-E' mNG-Rok (green in composites) and myosin regulatory light chain (labeled using *sqh-mCherry*; magenta in composites) colocalise in their accumulation across embryonic tissues: **B-C'** salivary gland placode, **D-D'** posterior spiracles, **E-E'** stage 15 epidermis.

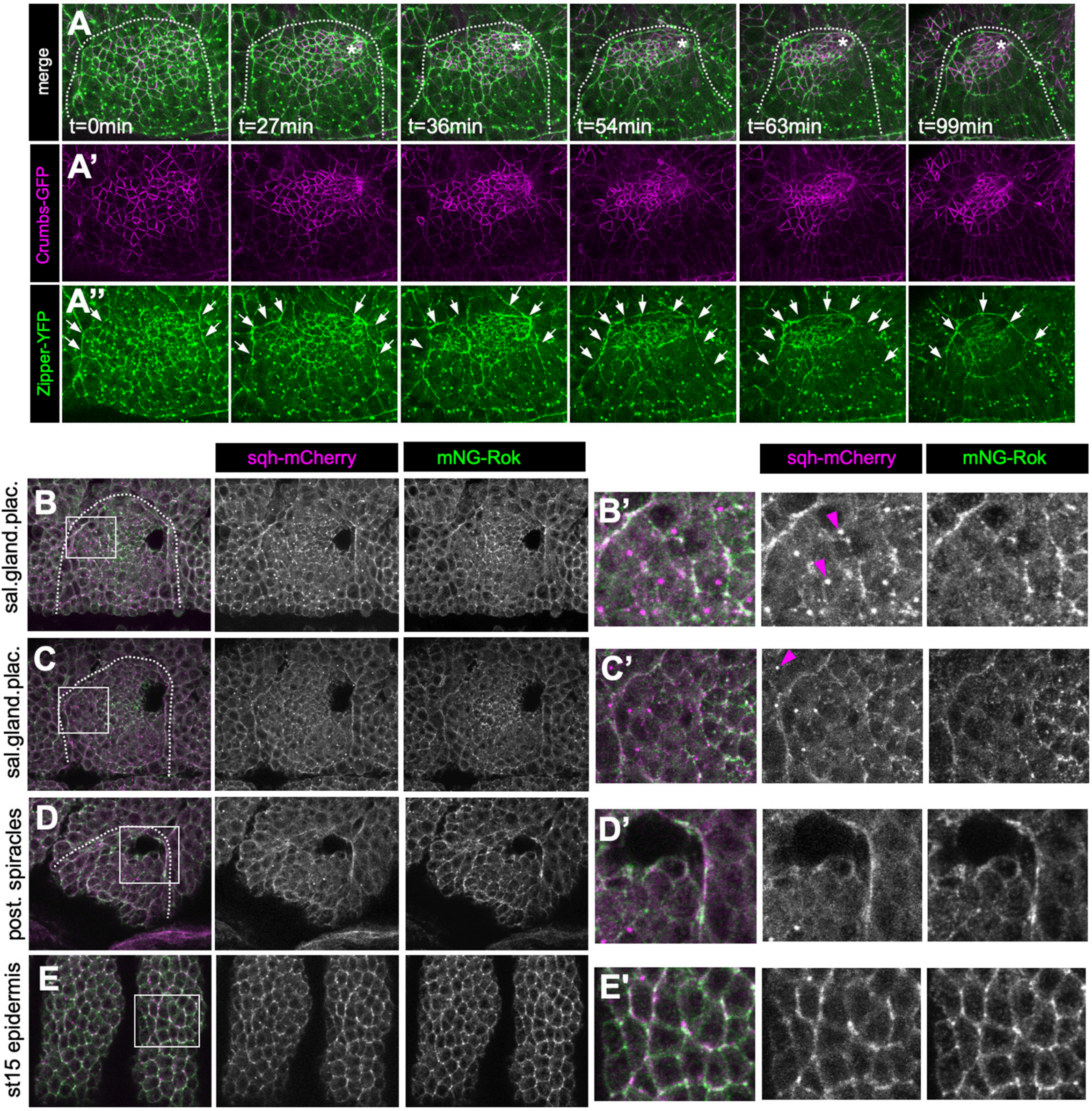

Sidor et al._Figure S1

Figure S2, related to Figure 2. Crumbs membrane localisation locally affects Rok membrane localisation.

Overexpression of Crumbs in stripes (white brackets and magenta arrows), using *en-Gal4 x UAS-Crumbs*, within the epidermis leads to spreading of Crumbs (magenta in **A-C**) to more basal positions along the lateral plasma membrane. This in turn leads to a basal displacement of mNG-Rok (green) along the lateral sides, visible through higher mNG-Rok membrane levels at a sub-apical position (**B',C'**, white arrows indicate mNG-Rok position) and loss of it at the apical-most lateral position (**A'**).

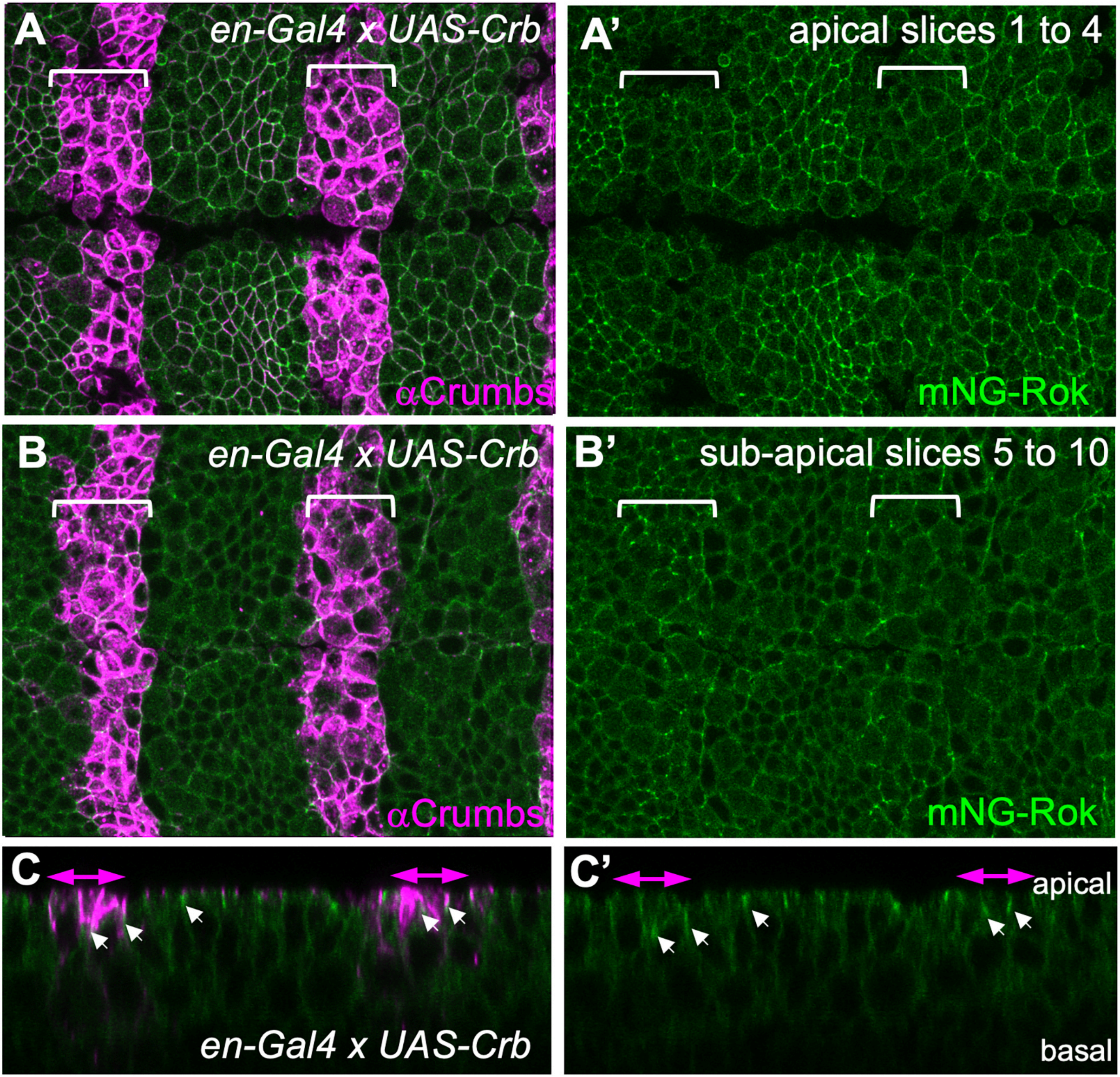

Sidor et al._ Figure S2

Figure S3, related to Figure 3. FRAP analysis and in silico simulation of Rok dynamics at the placode boundary versus inside the placode.

A Membrane versus cytoplasm enrichment of mNG-Rok in inner placodal cells (2.37) and surrounding epidermal cells (2.64). Data are represented as data points, mean and SEM. **B** Comparison of mNG-Rok levels in different membranes (membrane illustrated in the box), expressed as ratios of different membrane domains: boundary vs side (1.41), boundary vs inner (1.22), side vs inner (0.79), inner vs outer (1.59).

C Mobile fraction of mNG-Rok at placode boundary membranes (blue; value is 66%), side membranes (magenta; value is 92%) or inner membranes (orange; value is 77%), data points, mean and spread are shown. **D** Scatter plot of k_{off} versus mobile fraction for boundary vs side and inner junctions, showing close clustering of side and inner and clear difference to boundary junctions.

E Outputs of *in silico* simulations as in Figure 3G, comparing the same combinations of k_{off} values paired with different k_{on} values (0.2; 0.3; 0.4). Color code for particle enrichment at junctions as in Figure 3G, numbers on representative junctions are the particle numbers derived from simulations.

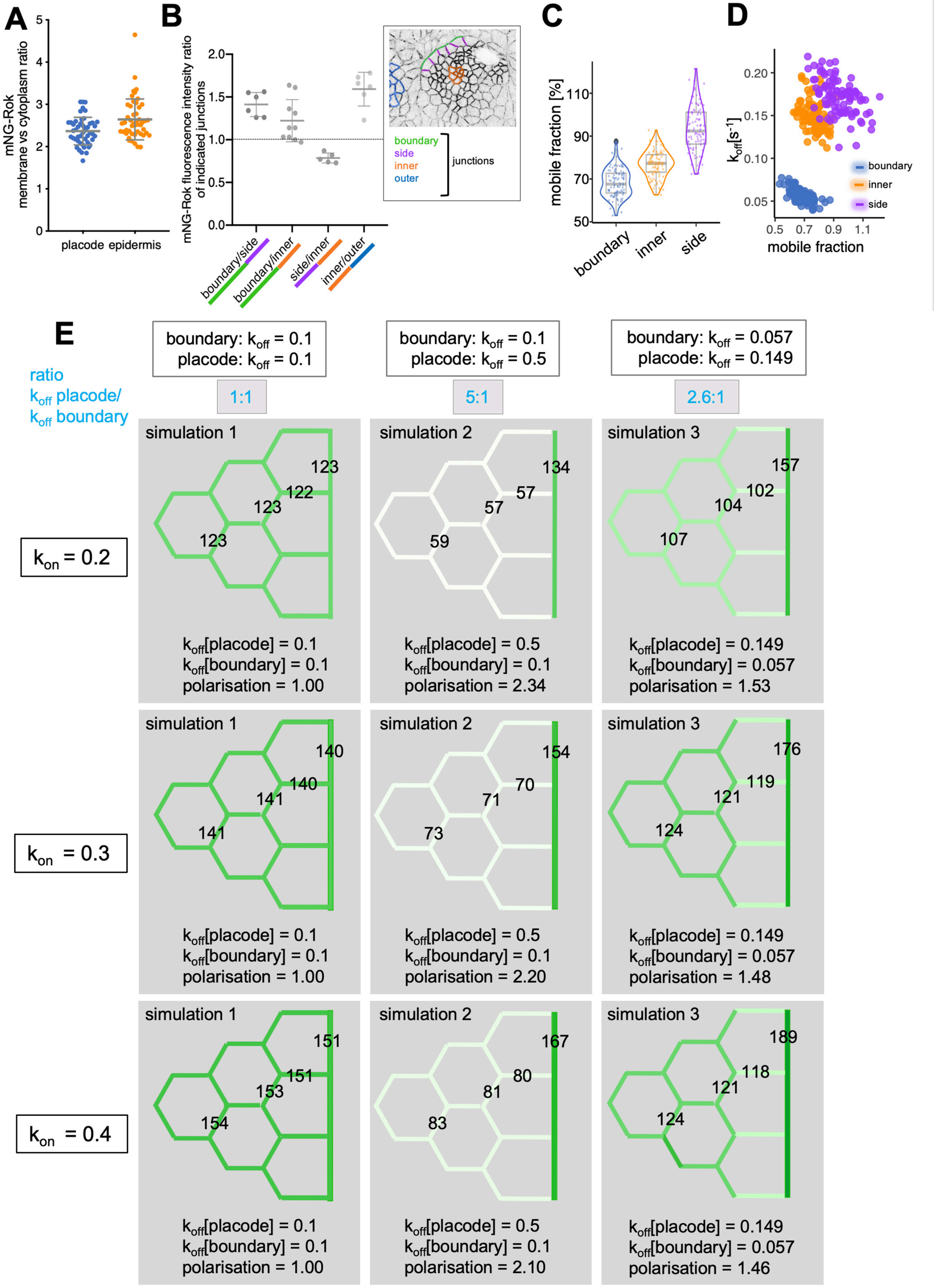

Sidor et al._Figure S3

Figure S4, related to Figure 5. Analysis of predicted aPKC and Pak1 phosphorylation sites in Dm Rok.

We used published predictive algorithms to identify potential aPKC and Pak1 phosphorylation sites in *Dm* Rok. Residues marked in yellow are predicted Pak1 sites (using DISPHOS) with a score >0.8, grey residues are predicted Pak1 sites (using DISPHOS) with a score between 0.6 and 0.8. Blue marks residues as predicted aPKC target sites (using NetPhos3.1). Green residues are predicted target sites for both aPKC and Pak1. Bold, underlined residues mark the RBD and PH domain. Conserved residues between human ROCK1 and *Dm* Rok that were found to be phosphorylated in EpH4 cells are shown in red and underlined (Ishiuchi and Takeichi, 2011).

***Dm* Rho-kinase (isoform PA)**

MPAGRETVTK QRSMDVERRR RANTLEREMR DPTSICNVDC LLDTVSALVS DCDHESLRRL
KNIEQYAAKY KPLAMQINQL RMNVEDFHFI KLIGAGAFGE VQLVRHKSSS QVYAMKRLSK
FEMMKRPDSA FFWEERHIMA HANSEWIVQL HFAFQDAKYL YMVMDFMPGG DIVSLMGDYD
IPEKWAIFYT MEVVLALDTI HNMGFVHRDV KPDNMLLDSY GHLKLADFGT CMRMGANGQV
VSSNAVGTPD YISPEVLQSQ GVDNEYGREC DWWSVGIFLY EMLFGETPFY ADSLVGTYGK
IMDHKNSLSF PPEVEISEQA KALIRAFLTD RTQRLGRYGI EDIKAHPFFR NDTWSFDNIR
ESVPPVVPEL SSDDDTRNFE DIERDEKPEE VFPVPKGFDG NHLPFIGFTY TGDYQLLSSD
TVDAESKEAN VANSGAASNN HGHGHNHRHR PSNSNELKRL EALLERERGR SEALEQQDAG
LRQQIELITK REAELQRIAS EYEKDLALRQ HNYKVAMQKV EQEIELRKKT EALLVETQRN
LENEQKTRAR DLNINDKVVS LEKQLLEMEQ SYKTETENTQ KLKKHNAELD FTVKSQEEKV
RDMVDMIDTL QKHKEELGQE NAELQALVVQ EKNLRSQLKE MHKEAENKMQ TLINDIERTM
CREQKAQEDN RALLEKISDL EKAHAGLDFE LKAAQGRYQQ EVKAHQETEK SRLVSREEAN
LQEVKALQSK LNEEKSARIK ADQHSQEKER QLSMLSVDYR QIQLRLQKLE GECRQESEKV
AALQSQLDQE HSKRNALLSE LSLHSSEVAH LRSRENQLQK ELSTQREAKR RFEEDLTQLK
STHHEALANN RELQAQLEAE QCFSRLYKTQ ANENREESAE RLSKIEDLEE ERVSLKHQVQ
VAVARADSEA LARSIAEETV ADLEKEKTIK ELELKDFVMK HRNEINAKEA ALATLKEAEN
ELHKKLGQKA AEYEDLVQQH KKQQEELALM RSSKDEEITK LLDKCKNEVL LKQVAVNKLA
EVMNRRDSDL PKQKNKARST AELRKKEKEM RRLQQELSQE RDKFNQLLLK HQDLQQLCAE
EQQLKQKMVM EIDCKATEIE NLQSKLNETA SLSSADNDPE DSQHSSLLSL TQD**SVFEGWL**
SVPNKQNRRR GHGWKRQYVI VSSRKIIFYN SDIDKHNTTD AVLILDLSKV YHVRSVTQGD
VIRADAKEIP RIFQLLYAGE GASHRPDEQS QLDVSVLHGN CNEERPGTIV HKGHEFVHIT
YHMPTACEVC PKPLWHMFKP PAAYECKRCR NKIHKEHVDK HDPLAPCKLN HDPRSARDML
LLAATPEDQS LWVARLLKRI QKSGYKAASY NNNSTDGSKI SPSQSTRSSY KPYAVNVQRS
ATLPANSSLK

→ predicted Pak1 sites score>0.8 (DISPHOS results)
→ predicted Pak1 sites 0.6<score<0.8 (DISPHOS results)
→ predicted aPKC sites (NetPhos3.1)
→ putative sites for both aPKC and Pak1
→ peptides tested in vitro
→ conserved residues between human and *Drosophila* that are phosphorylated in EpH4 cells
→ **bold and underlined** residues mark the RBD and PH domain

Sidor et al._Figure S4

Figure S5, related to Figure 7. mNG-Rok dynamics under Pak1-level modulation.

A Mobile fraction of mNG-Rok at placode boundary membranes and inner membranes when Pak1 levels are modulated: *UAS-Pak1*^{myr}/boundary (dark blue; value is 87%), *UAS-Pak1*^{myr}/inner(orange; value is 71%), *pak1*^{14}/boundary (light blue; value is 58%), *pak1*^{14}/inner (light brown; value is 70%). Data points, mean and spread are shown. **B** Scatter plot of k_{off} versus mobile fraction for boundary vs inner junctions when Pak1 levels are modulated, showing close clustering of *UAS-Pak1*^{myr} for both boundary and inner junctions, as well as the overlap for the *pak1*^{14} mutant for both boundary and inner junctions.

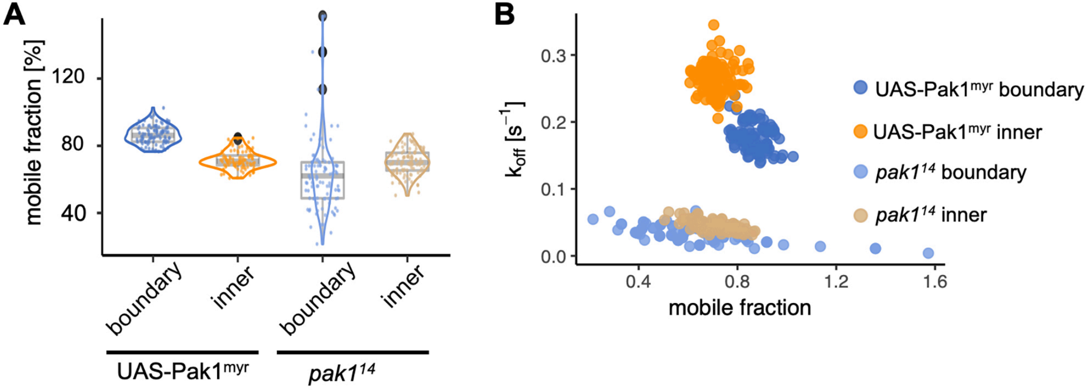

Sidor et al._Figure S5
